# Influence of the hard segments content on the properties of electrospun aliphatic poly(carbonate-urethane-urea)s†

**DOI:** 10.1039/d4ra01726a

**Published:** 2024-05-15

**Authors:** Karolina Rolińska, Hadi Bakhshi, Maria Balk, Paweł Parzuchowski, Magdalena Mazurek-Budzyńska

**Affiliations:** a Faculty of Chemistry, Warsaw University of Technology Noakowskiego 3 00-664 Warsaw Poland magdalena.budzynska@pw.edu.pl; b Faculty of Chemistry, University of Warsaw Pasteura 1 02-093 Warsaw Poland; c Łukasiewicz Research Network – Industrial Chemistry Institute Rydygiera 8 01-793 Warsaw Poland; d Department of Life Science and Bioprocesses, Fraunhofer Institute for Applied Polymer Research IAP Geiselbergstraße 69 14476 Potsdam Germany; e Institute of Active Polymers, Helmholtz-Zentrum Hereon Kantstraße 55 14513 Teltow Germany

## Abstract

The study investigated the impact of hard segments (HS) content on the morphology and thermomechanical properties of electrospun aliphatic poly(carbonate-urea-urethane)s (PCUUs). The obtained nonwovens exhibited surface porosity ranging from 50% to 57%, and fiber diameters between 0.59 and 0.71 μm. Notably, the PCUUs nonwovens with the highest HS content (18%) displayed superior mechanical properties compared to those with lower HS contents. This study highlights the ability to customize the properties of polymeric nonwovens based on their chemical compositions, offering tailored solutions for specific application needs.

## Abstract 2

The study investigated the impact of hard segments (HS) content on the morphology and thermomechanical properties of electrospun aliphatic poly(carbonate-urea-urethane)s (PCUUs).

## Graphical abstract

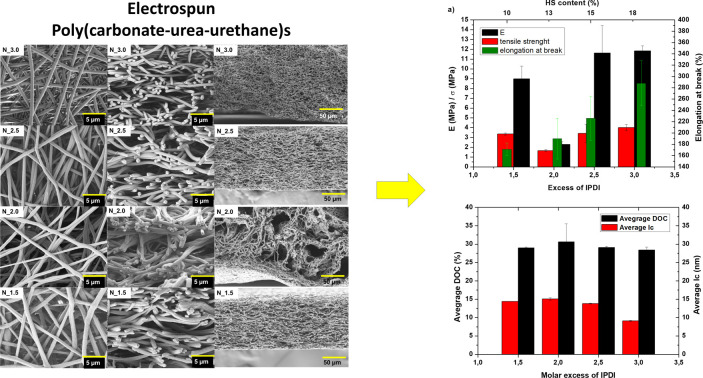

## 1. Introduction

The soaring demand and pervasive acclaim for polyurethanes (PUs) are rooted in their extraordinary physical and mechanical attributes, encompassing unmatched resilience, flexibility, and resistance to abrasion. These versatile materials play a pivotal role in shaping not only flexible and rigid foams, but also find applications in adhesives, elastomers, fibers, gaskets, and high-performance coatings.^1–3^

Conventional PUs are typically synthesized using polyisocyanates, polyols, and chain extenders. PUs typically occur in the form of multi-block copolymers, where distinct phases occur due to the presence of two types of segments within the chains: rigid (hard) and elastic (soft). The soft segments, primarily composed of linear polyols, contrast with the rigid segments formed by derivatives of isocyanate groups and chain extenders. The materials' properties hinge on the molecular configuration of these segments and their relative molar ratios.

The versatile nature of PUs arises from the ability to tailor their properties by carefully selecting the reacting components. Polyester diols, when used as soft segments in PUs, contribute to polymers with commendable physical properties. However, these formulations are prone to hydrolytic degradation due to the susceptibility of ester linkages to hydrolysis.^4^ In contrast, PUs based on polyether diols offer enhanced resistance to hydrolysis, making them preferable in applications where hydrolytic stability is crucial. Nonetheless, poly(ether-urethane)s are susceptible to oxidation processes. To address these drawbacks, a promising solution involves replacing the polyester or polyether segments with polycarbonate counterparts.^5,6^ This strategic substitution not only overcomes the susceptibility to hydrolytic degradation seen in poly(ester-urethane)s but also mitigates the oxidation vulnerability observed in poly(ether-urethane)s.^7^ These attributes make poly(carbonate-urethane)s (PCUs) highly desirable as biomaterials for enduring long-lasting implantation applications, such as in the spine, as meniscus implants, or as components for artificial hearts.^8–14^

PCUs stand out not only for their exceptional mechanical and biological properties but also for being an environmentally friendly starting material. The conventional method of production of PCUs utilizes soft segment precursors known as oligocarbonate diols (OCDs). OCDs can be obtained from dimethyl carbonate (DMC) at a low reaction temperature, specifically at the boiling point of the DMC/methanol azeotrope (64 °C).^15,16^ This approach offers the added benefit of conducting the reaction without the need for additional organic solvents. The incorporation of carbon dioxide in the synthesis of OCDs enhances their environmental credentials, positioning them as environmentally friendly “green starting materials.”

Electrospinning (ES) is one of the methods, which enables the creation of fibrous structures similar to the natural extracellular matrix.^17^ The application of the ES technique for manufacturing fibers compared to traditional spinning methods is distinguished by the smaller diameter of the obtained fibers. ES allows obtaining uniform fibers in the diameter range in micro- and nanometers.^18^ Electrospun nonwovens are often used as dressing materials due to their good barrier properties and oxygen permeability.^19–22^ PUs are often employed to produce nanofibers due to their chemical stability and excellent fiber-forming properties.^23^ PU nonwovens have been applied in *e.g.* high-efficiency air filters, protective textiles, dressing materials, sensors, and drug carriers.^20–22^ PCU fibers can also be obtained using the ES technique,^24,25^ especially for application in tissue engineering.^26^ For example, PCU nonwovens have been used for fabricating vascular grafts (VGs).^27–31^ Recently, a green chemistry approach was utilized to obtain high-molecular-weight non-isocyanate polyurethanes (NIPUs) based on polycarbonate diols, which can be processed by ES process and have shown good adhesion of fibroblasts and epithelial cells.^32^ A polyhedral oligomeric silsesquioxane poly(carbonate-urea-urethane) (POSS–PCUU) was also utilized in the ES process and provided appropriate surface and mechanical properties for the fabrication of small-diameter vascular grafts with a single-layer endothelial barrier at the luminal surface.^33–35^ Furthermore, a PCUU called MyoLink™ was reported to be a good candidate for tissue engineering purposes.^36–41^

Recently, we have focused on the potential use of PCUU in the tissue engineering field. In a previous study^42^ we analyzed the chemical composition of PCUUs and selected the soluble polymer structures. Furthermore, we investigated the influence of the ES solution concentration on the thermal, mechanical, and biological properties of electrospun PCUU nonwovens.^43^ Here, we have investigated the effect of the PCUU structure, *i.e.* hard segments (HS) content, on the morphology and thermomechanical parameters of the obtained PCUU nonwovens.

## 2. Experimental

### 2.1. Materials

Tetrahydrofuran (THF, purity ≥ 99%) and *N*,*N*-dimethylformamide, (DMF, purity ≥ 99%) were purchased from POCH (Gliwice, Poland). Isophorone diisocyanate (IPDI, purity ≥ 98%) was purchased from Sigma-Aldrich (Poznań, Poland). Materials were used without any further purification. Oligo(decamethylene carbonate)diol (OCD) was synthesized according to the literature^42^ The detailed procedure for the synthesis of OCD is provided in ESI.† The synthesized OCD was characterized using ^1^H NMR and FTIR spectroscopy (Fig. S1 and S2 in ESI†). The molecular weight (*M*_n_) of OCD determined from the ^1^H NMR spectrum was equal to 3000 g mol^−1^.

### 2.2. Synthesis of PCUUs

The synthesis of PCUUs was performed according to the prepolymer method; in the first step, carbonate–urethane prepolymers were synthesized and in the second step, further chain-extended with water. The carbonate–urethane prepolymers were obtained accordingly: a total of 20.00 ± 0.15 g of OCD was placed in the reaction flask equipped with a thermometer and mechanical stirrer, and it was dried under reduced pressure at 90 °C for 1.5 h. Afterwards, IPDI was added in various molar ratios relative to OCD (OCD/IPDI: 1/1.5, 1/2, 1/2.5, and 1/3) and the reaction was continued at 80 °C without solvent or catalyst (Table S1 in ESI†).^15^ The synthesized carbonate–urethane prepolymers were characterized through FTIR spectroscopy (Fig. S3–S6 in ESI†).

The chain-extension reaction of the carbonate–urethane prepolymers with water vapor was performed in an open glass mold (10 cm × 10 cm) placed in a climatic chamber at 75 °C and 5% relative humidity for 1 day, then at 70 °C and 10% relative humidity for a further 4 days, and continuing at 60 °C and 40% relative humidity for 2 days. These reaction conditions were optimized to minimize the gelation/crosslinking phenomenon. The progress of the chain-extension reaction was controlled through FTIR spectroscopy to observe the disappearance of the peak of the isocyanate (NCO) group at 2260 cm^−1^. Filtration trials to remove the possibly generated gel/crosslinked parts were not successful and the obtained PCUUs were used without further purification. The synthesized PCUUs were named accordingly: PCUU_X where X means the molar ratio of IPDI to OCD used for the synthesis. For example, PCUU_1.5 means that the PCUU was obtained from OCD and IPDI in the molar ratio of 1/1.5.

The synthesized PCUUs were characterized through ^1^H NMR and FTIR spectroscopy (Fig. S7–S15 in ESI†). The molecular weights of synthesized PCUUs were determined by means of GPC (Table 1 and Fig. S16–S19 in ESI†).

**Table 1:** The molecular weight of PCUUs determined by means of GPC

Sample	HS (wt%)	*M*_n_ (g mol^−1^)	*M*_w_ (g mol^−1^)	DI
PCUU_3.0	18 ± 1	23 600	2 008 000	85.3
PCUU_2.5	15 ± 1	56 300	1 674 000	29.7
PCUU_2.0	13 ± 1	43 800	2 665 000	60.9
PCUU_1.5	10 ± 1	64 600	1 169 000	18.1

### 2.3. ES process

PCUUs were dissolved in DMF/THF mixture (50/50, wt/wt) at a concentration of 4 wt% at room temperature by stirring for 72 h. The ES process was performed in the laboratory spinning unit equipped with a drum collector (MTI Corporation, model: MSK-ESDC-80-4000) with dimensions of 80 × 200 mm with a speed controller. The rotational speed of the drum collector was 150 rpm. The distance from the collector to the nozzle was 20 cm. Each solution was placed in a 20 ml syringe and electrospun on the collector (covered with aluminum foil) through a 22 G needle with a 0.41 mm inner diameter. The power supply was set up for a positive voltage of 18 kV. The flow rate of the solution was set up on the syringe pump at 1.5 ml h^−1^. The relative humidity and temperature values at the time of the experiments ranged from 40 to 52% and from 24 to 27 °C, respectively. The obtained nonwovens were named accordingly: N_X where X means the molar excess of the IPDI used for the synthesis of PCUUs. For example, N_1.5 means that the electrospun mat was obtained from 4 wt% solution of PCUU synthesized using OCD (*M*_n_ = 3000 g mol^−1^) and IPDI in the molar ratio of 1/1.5.

### 2.4. Instruments and methods

^1^H-NMR spectra were recorded at 298 K on a Varian VXR 400 MHz Spectrometer (Palo Alto, CA, USA) using tetramethylsilane as an internal reference and CDCl_3_ as a solvent and were analyzed with MestReNova v.6.2.0-7238 (Mestrelab Research S.L) software. The error of the method was estimated based on the error of the integral peak area (around 5%).

ATR-FTIR spectra were recorded on a Thermo Scientific Nicolet iS5 FTIR spectrometer using an ATR iD7 accessory. 32 scans were recorded for each sample.

The gel permeation chromatography (GPC) measurements were performed using a Malvern ViscotekGPCMax TDA 305 apparatus, equipped with a Jordi Labs DVD Mixed Bed column of 30 cm long and with an internal diameter of 7.8 mm. The apparatus had four detectors: refractometric, light scattering, viscometric, and UV-PDA. The measurement was carried out at a temperature of 30 °C and with an eluent flow (DCM) of 1 ml min^−1^. The apparatus was calibrated using sharp PS standards. Approximately 2–3 mg of the solid sample was dissolved in 1.5 ml of DCM with the addition of 1% vol. CHCl_3_. After complete dissolution, the solutions were passed through a syringe filter with a 0.2 μm PTFE membrane.

The surface morphology of nonwovens was investigated by SEM measurements, where samples were cut using sharp razor blades and stuck on specific holders with conductive adhesive. The samples were sputtered with gold achieving a thickness of 5 nm. Samples were then investigated with a desktop-SEM Phenom G2 from PhenomWorld (LOT-Oriel Group Europe, Darmstadt, Germany). To investigate the cross-section, samples were moistened with isopropanol, cooled with liquid nitrogen, broken by a blade, and stuck on specific holders with conductive adhesive. The samples were sputtered with iridium achieving a thickness of 4 nm and were investigated with a SEM Supra 40VP (Carl Zeiss Company, Oberkochen, Germany).

The average fiber diameter and surface porosity of the electrospun samples was calculated from SEM images of the top sides at a magnification of 5000 and 500 using ImageJ software (version 1.52p). The reported values were calculated based on three batches of electrospun nonwovens.

Differential scanning calorimetry (DSC) was performed on Netzsch DSC 204 (Netzsch Ltd Selb. Germany) in sealed Al-pans under N_2_-atmosphere between −70 and 150 °C with heating and cooling rates of 10 °C min^−1^.

Wide angle X-ray scattering (WAXS) measurements were conducted at ambient temperature in transmission geometry utilizing the X-ray diffraction system Bruker D8 Discover (generator operated at 40 kV and 40 mA) with a two-dimensional detector from Bruker AXS (Karlsruhe. Germany). The X-ray beam (Cu-Kα1-radiation. *λ* = 0.154 nm) was provided by a graphite monochromator and a pinhole collimator with an opening of 0.8 mm. Sample-to-detector distance was 15 cm applying an irradiation time of 120 seconds. Integration of the 2-D intensities gave linear intensity curves *I* (2*θ*). Crystallinity values were calculated as an average of three individual fits of the scattering curve with Pearson 7 functions utilizing TOPAS(R) software from Bruker AXS.

Dynamic mechanical thermal analysis (DMTA) measurements were performed on Eplexor 25 N (Gabo. Ahlden. Germany) equipped with a 25 N load cell using the standard type test specimen (DIN EN ISO 527-2/1BB). The applied oscillation frequency was 1 Hz. The measurements were performed in the temperature sweep mode from −100 to 150 °C with a constant heating rate of 3 °C min^−1^.

Tensile tests were conducted with standard samples (ISO 527-2/1BB) cut from mats on a tensile tester Z75 (Zwick, Ulm, Germany) equipped with thermo-chamber (Mytron Bio-und Solartechnik, Heilbad Heiligenstadt, Germany), temperature controller Eurotherm control 2408 (Eurotherm Regler, Limburg, Germany), and load cells suitable to determine maximum forces of 200 N (Zwick, Ulm, Germany). The strain rate in the uniaxial tensile test was 10 mm min^−1^. The average value of the tensile strength (*σ*), elongation at break (*ε*), and Young's modulus (*E*) for each type of material were determined from five specimens. Measurements were performed at room temperature and 37 °C.

## 3. Results and discussion

### 3.1. Synthesis and ES of PCUUs

**Scheme 1:**
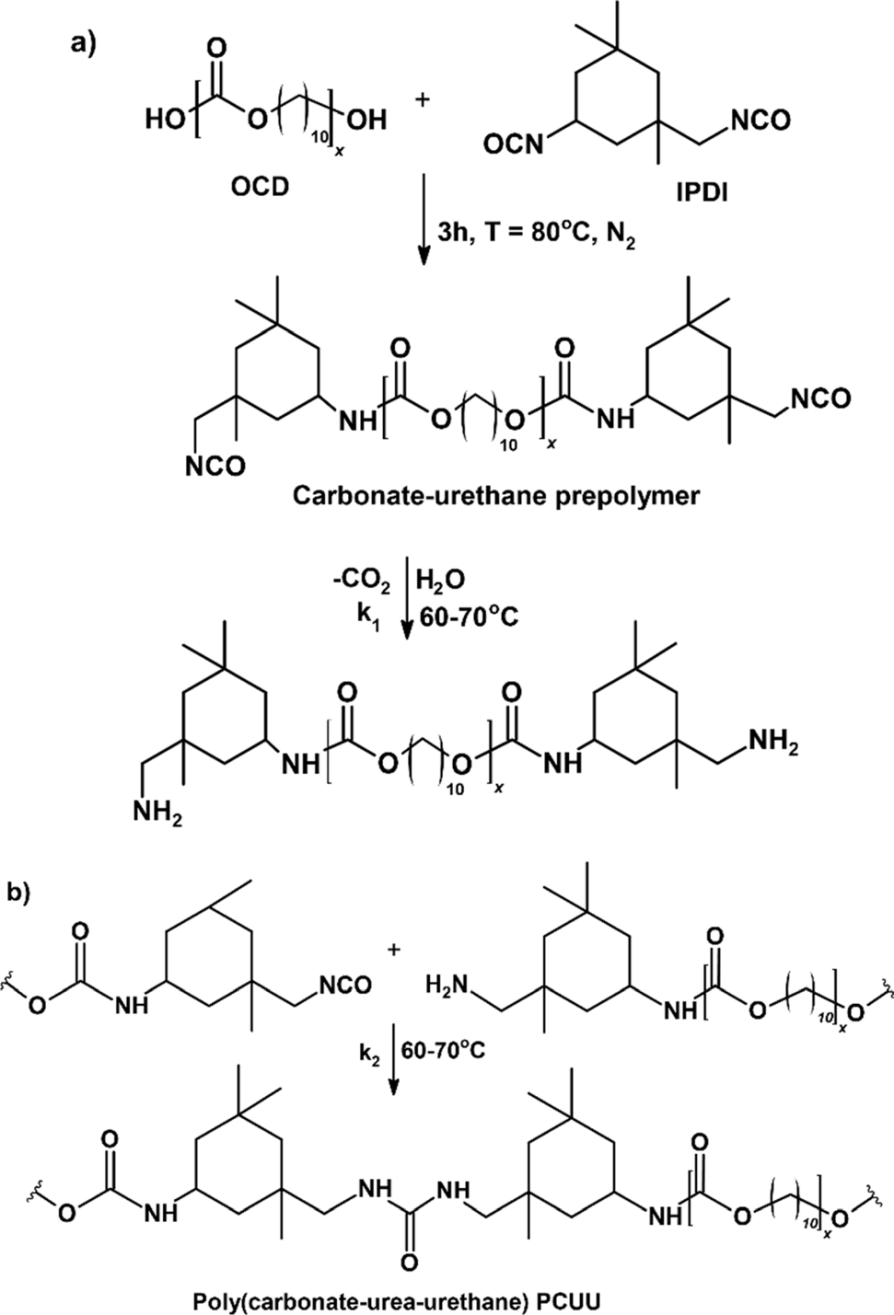
The synthesis of PCUUs; (a) synthesis of carbonate–urethane prepolymer and its' hydrolysis, (b) formation of urea bonds in PCUU.

The primary objective of this study was to investigate the applicability of PCUUs for the ES process and to assess the resulting nonwovens. Our focus was on evaluating how variations of HS content in PCUUs could influence the properties of the corresponding nonwovens. PCUUs were obtained using OCD as polyol according to the prepolymer method based on IPDI and water as a chain-extension precursor (Scheme 1).^6,44–47^ Four various molar ratios of OCD/IPDI were used (1/1.5, 1/2, 1/2.5, and 1/3).^42^ The reaction was carried out without solvent or catalyst. The obtained isocyanate prepolymers were poured into an open glass mold (10 cm × 10 cm) and placed in a climatic chamber, in which the chain-extension reaction proceeded under controlled conditions of humidity (5–40%) and temperature (60–70 °C). Water vapors were used to hydrolyze the isocyanate to amine groups (Scheme 1a). Following reaction of the amine with diisocyanate groups resulted in polymer chain-extension whereby urea groups were formed (Scheme 1b). Importantly, the reaction rate constant for the hydrolysis of isocyanate groups (*k*_1_) is much slower than reaction rate constant for the urea formation (*k*_2_). Therefore, stoichiometry of the reaction is controlled and chain-extension progress is provided.

The chain-extension reaction conditions were optimized to minimize the gelation/crosslinking phenomenon. More details about this procedure can be found in ref. 42. Crosslinking can occur through the reaction of isocyanate groups with either urethane or urea bonds, resulting in allophanate and biuret groups, respectively, which are possible in the presence of catalysts and at temperatures higher than 90 °C. Filtration trials to remove the possibly generated gel/crosslinked parts were not successful and the obtained PCUUs were used without further purification.

The progress of the chain-extension reaction was controlled by means of the FTIR spectroscopy and carried out until the disappearance of the signal assigned to stretching vibration of NCO groups at 2260 cm^−1^. The amounts of reagents used are summarized in Table S1 in ESI.†^1^H NMR spectra of the obtained PCUUs are shown in Fig. S7–S10 in ESI.† FTIR spectra of the obtained PCUUs are shown in Fig. S11–S15 in ESI.† The molecular weights of the PCUUs were determined using GPC (Table 1 and Fig. S16–S19 in ESI†).

**Fig. 1:**
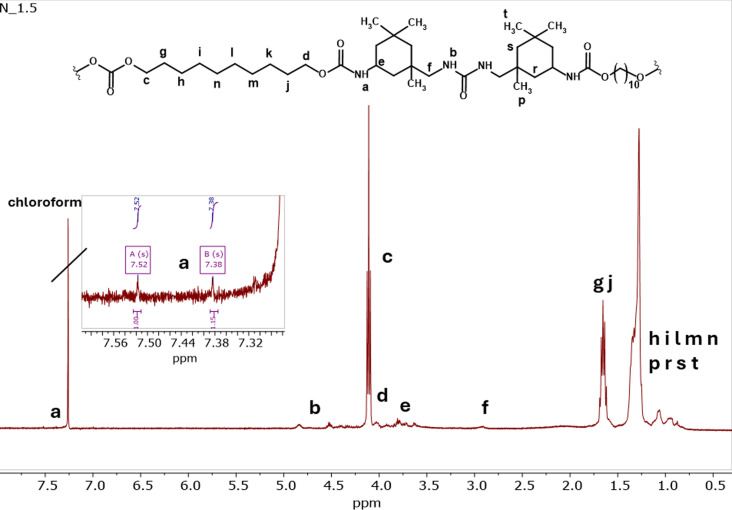
^1^H NMR (CDCl_3_, 400 MHz) spectra of N_1.5.

Electrospun nonwovens were also characterized by means of FTIR (Fig. S20 in ESI†) and NMR spectroscopy (Fig. 1). Content of HS in PCUUs is relatively low (10–18%), therefore signals originating from urethane at 7.0–8.0 pm (‘a’) and of urea groups at 4.5–5.0 ppm (‘b’) in ^1^H NMR spectra have very low intensity in comparison to signals originating from soft segments and are too small for complex analysis and interpretation. However, due to two types of amine groups in the IPDI structure, two types of signals originating from urethane and urea groups in PCUU are visible in spectra. The characteristic signals for C*H*_2_–OC(O)O in the direct connection with carbonate group were observed at 4.1 ppm (signals ‘c’) and at 4.0 ppm (signals ‘d’) C*H*_2_–O(O)C–NH- proton connected with urethane group are present. At 3.8–3.6 ppm (signals ‘e’) signals of protons of C*H*_2_–NH–C(O)O- in the direct connection with of urethane group, and at around 3.0 ppm (signals ‘f’) CH_2_- in the direct connection with –NH– in the urea group were detected.

### 3.2. Morphology of PCUU nonwovens

**Fig. 2:**
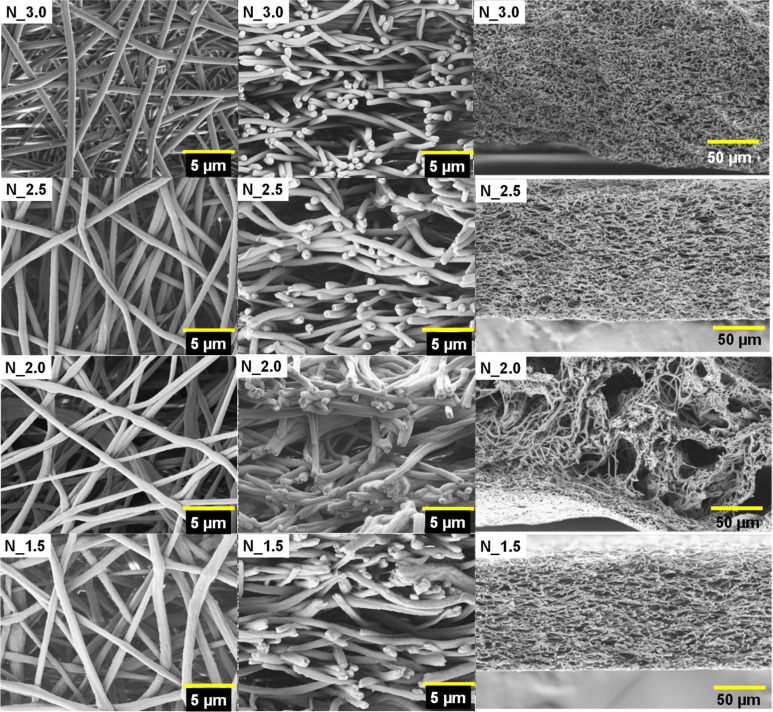
SEM micrographs of the surface (left) and cross-section (middle and right) of PCUU nonwovens.

Morphology is one of the most essential components characterizing electrospun nonwovens. It depends on many factors, such as the polymer and solvent types as well as ES process parameters. The SEM micrographs for electrospun PCUU nonwovens are collected in Fig. 2. The average fiber diameter and surface porosity extracted from SEM micrographs are presented in Table 2. Based on the results, it can be concluded that the synthesized PCUUs are electrospinnable and can be used to obtain uniform fibers. However, in the case of N_1.5 and N_2.0, surface irregularities in fibers were observed. We observed some crosslinked parts in the corresponding PCUU solutions. These crosslinked parts can result in fragments within fibers with bigger diameters. It is worth mentioning that due to the same reason, N_1.5 and N_2.0 presented higher standard deviation values for the average fiber diameter (Table 2).

**Table 2:** The morphological properties of PCUU nonwovens determined by SEM

Sample	Average fiber diameter (μm)	Surface porosity (%)
N_3.0	0.50 ± 0.09	57.4 ± 1.5
N_2.5	0.59 ± 0.08	54.1 ± 1.1
N_2.0	0.60 ± 0.16	55.0 ± 6.7
N_1.5	0.71 ± 0.18	50.4 ± 2.8

The diameters of the fibers increased with the decrease in HS content and were 0.50 ± 0.09 in the case of N_3.0 and 0.71 ± 0.18 μm in the case of N_1.5 (Table 2). Moreover, the surface porosity of the nonwovens decreased with the increase of the fiber diameter from 57.4 ± 1.5% in the case of N_3.0 to 50.4 ± 2.8% in the case of N_1.5. The fiber diameter could be affected by the molecular weight of PCCUs. We have observed that in the case of the highest *M*_n_ of the PCUU, the average fiber diameter was the largest. This phenomenon follows the general rule that high molecular weight polymers give more viscose solutions, slower jetting, and consequently fibers with larger average diameters.^48,49^ Dong *et al.* reported that the average diameter of the electrospun PS fibers increased with increasing polymer molecular weight.^50^ Similarly, it was shown that the measured fiber diameter of PVA not only increased across polymer concentrations but also with the increase to the higher molecular weight.^51^ In the case of N_2.0 and N_2.5 the values of fiber diameter and surface porosity were similar. However, N_2.0 was characterized by an inhomogeneous structure with very big pores/holes (Fig. 2). We observed some crosslinked parts in the corresponding PCUU_2.0 solution, which may be attributed to this phenomenon.

### 3.3. Crystallinity of PCUU nonwovens

The WAXS analysis was performed to assess the impact of varying IPDI excess on PCUU structures. This analysis is particularly complex in the context of electrospun nonwovens due to several key factors. ES processes yield intricate fibrous structures, where the arrangement of polymer chains plays a pivotal role. Additionally, WAXS can serve as a critical tool to ensure the consistency and quality of the electrospun nonwovens by confirming whether the desired structural properties have been achieved. Moreover, understanding the structural characteristics through WAXS analysis enables researchers to establish essential correlations between the structure and properties of electrospun nonwovens, which is vital for optimizing their performance in various applications.

**Fig. 3:**
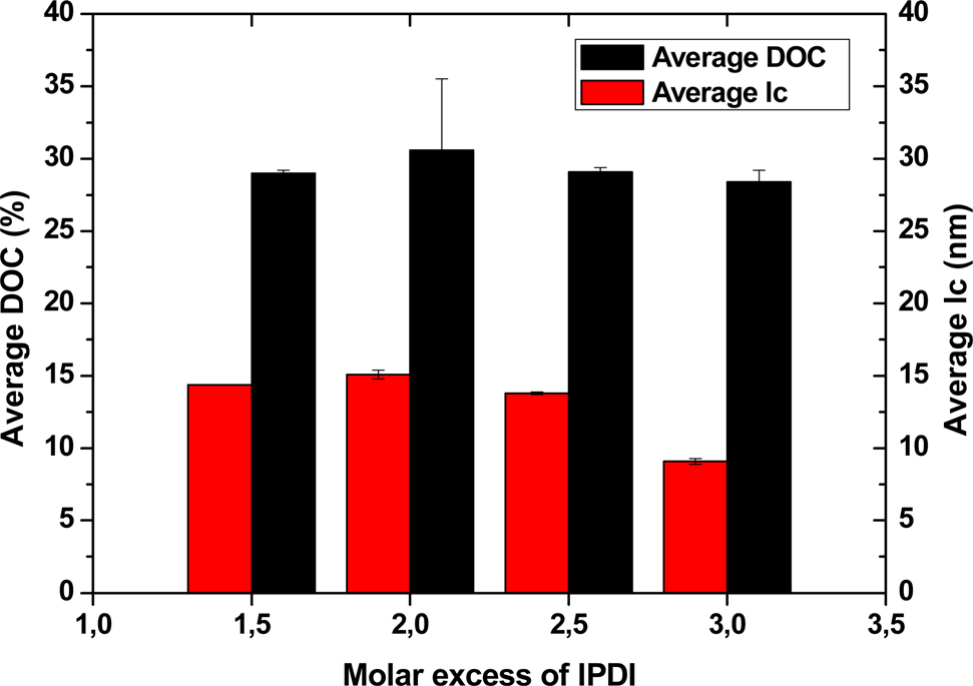
Average degree of crystallinity and average crystal size of PCUU nonwovens determined by WAXS.

There was no significant difference in the WAXS patterns among different IPDI excess levels in the PCUU structures (Fig. S21 in ESI†). Signals observed in WAXS spectra were assigned only to crystalline phase of soft segments (OCDs). The average degree of crystallinity (DOC) was 28.4 ± 0.8% and 29.0 ± 0.2% for N_3.0 and N_1.5, respectively, whereas the average crystals size (*l*_c_) for N_3.0 and N_1.5 was 9.1 ± 0.2 nm and 14.4 ± 0.1 nm, respectively (Fig. 3, Table S3 in ESI†). The results suggest that higher IPDI excesses tend to yield less crystalline structures of OCDs, implying that increased IPDI content leads to the formation of less ordered and more amorphous polymer chains, which is a consequence of asymmetric structure of IPDI, which hinders the regular structure of PCUUs. The same conclusion can be stated based on DSC results (Table 3). With the increase of the HS content in PCUUs, melting enthalpy (Δ*H*_m_) decreased, which is correlated with an decrease of crystalline phase of nonwovens.

**Table 3:** Thermal properties of the soft segments of PCUU nonwovens determined by DSC

Sample	1st run		2nd run		*T*_c_ (°C)	Δ*H*_c_ (J g^−1^)
	*T*_m,1st_ (°C)	Δ*H*_m,1st_ (J g^−1^)	*T*_m,2nd_ (°C)	Δ*H*_m,2nd_ (J g^−1^)		
N_3.0	49 ± 1	52 ± 1	42 ± 1	23 ± 1	−9 ± 1	23 ± 1
N_2.5	48 ± 1	57 ± 1	45 ± 1	26 ± 1	−1 ± 1	28 ± 1
N_2.0	46 ± 1	62 ± 1	45 ± 1	25 ± 1	−2 ± 1	24 ± 1
N_1.5	47 ± 1	63 ± 1	49 ± 1	43 ± 1	13 ± 1	40 ± 1

### 3.4. Thermal properties of PCUU nonwovens

DSC was employed to investigate the thermal properties of electrospun of PCUU nonwovens, including melting (*T*_m_) and crystallization (*T*_c_) temperatures, and related enthalpies (Δ*H*_m_ and Δ*H*_c_). DSC analysis allows to characterize the thermal stability of material and its response to temperature changes, which is essential for determining their suitability for various processing and application conditions. Additionally, DSC provides valuable data on the impact of HS content on the thermal behavior of material, facilitating material design and selection. The results clearly demonstrated a pronounced correlation between the *T*_c_ and *T*_m_ values and the HS content within the PCUU structures – both, the *T*_c_ and *T*_m_ values (from the second heating curve in DSC) decreased with increasing the HS content of PCCU nonwovens (Table 3, and Fig. S22–S25†). The highest *T*_c_ of 12 °C was observed for N_1.5, with the lowest HS content among the PCUUs. It is related to the highest content of easily crystallizable oligo(decamethylene carbonate)s, which constitutes the soft segments. Additionally, most of the nonwovens exhibited elevated *T*_m,1st_ values (from the first heating curve in DSC) compared to *T*_m,2nd_ values (from the second heating curve in DSC), attributed to an increased proportion of the crystalline phase generated in the course of the ES process. For example, in the case of N_3.0 *T*_m,1st_ of 42 °C was observed, while for N_3.0 *T*_m,2nd_ took a value of 49 °C (Table 3). The stretching process between the needle and collector leads to the alignment of the polymer chains, consequently, resulting in an increase of the crystalline phase content (strain-induced crystallization).^52^ Therefore, also significantly higher values of Δ*H*_m,1st_ in comparison to Δ*H*_m,2nd_ were observed for example in case of N_3.0 from 23 to 53 (J g^−1^).

**Fig. 4:**
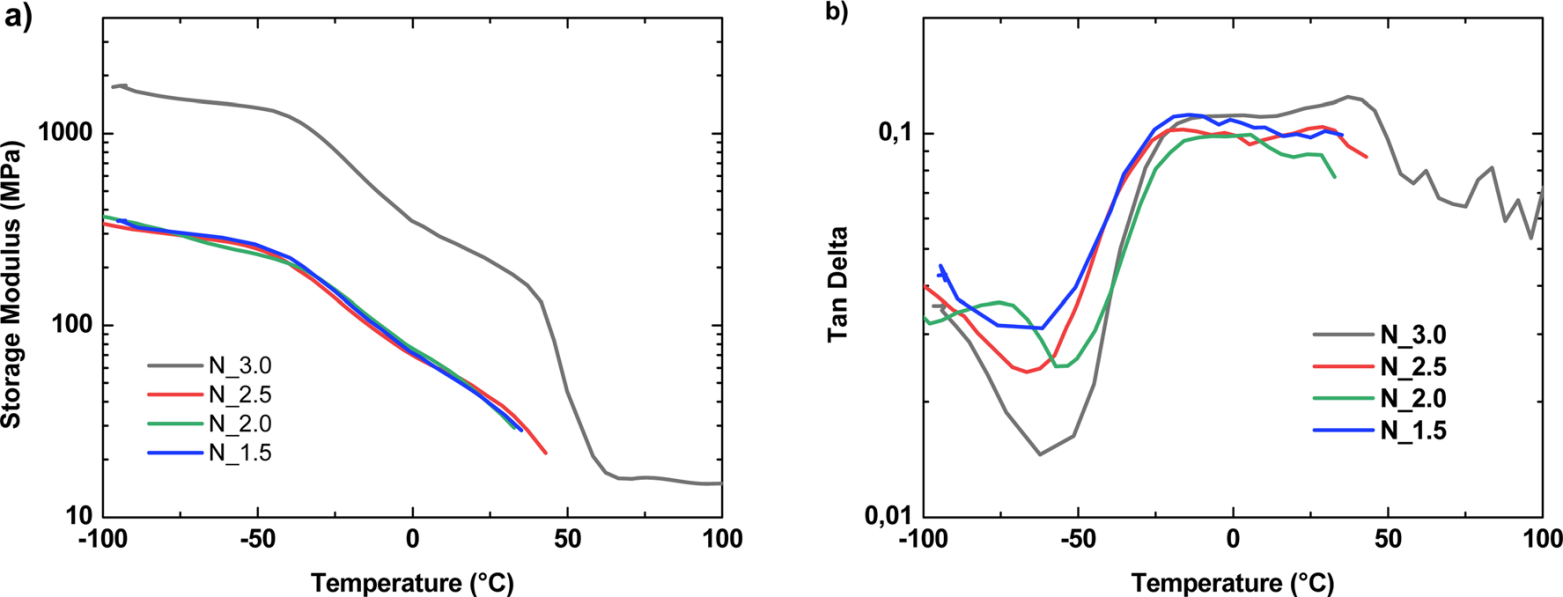
The storage modulus (a) and tan *δ* (b) as a function of temperature for PCUU nonwovens determined by DMTA.

Similarly, to the cast specimens, determining the glass transition temperature (*T*_g_) for nonwovens using conventional DSC was challenging.^42^ Hence, DMTA was employed for determining *T*_g_ of PCUU nonwovens (Fig. 4 and Table 4), due to its enhanced sensitivity to changes occurring during the glass transition compared to DSC, wherein the aforementioned thermal transition remained undetected. The *T*_g_ values determined from tan *δ* ranged between −11 and −16 °C with no clear trend. When determined by the storage modulus (*E*′), similar *T*_g_ values (within the margin of error) ranging between −12 °C and −15 °C were obtained. The *T*_m_ could only be determined for N_3.0 as other nonwovens broke during the heating procedure. The obtained *T*_m_ of soft segments (50 °C) was similar to the determined value by DSC (49 °C). Interestingly, nonwovens with lower HS content broke during the heating procedure related to the melting of soft segments, which indicates that N_3.0 obtained by 3 fold molar excess of IPDI was physically crosslinked by the high content of urea and urethane units. This could be also confirmed by the highest mechanical strength, and more than 10 times higher storage modulus of N_3.0 in comparison to other investigated nonwovens.

**Table 4:** Thermal properties of PCUU nonwovens determined by DMTA^a^

Sample	*T*_g_ (°C) from tan *δ*	*T*_g_ (°C) from *E*′	*T*_m_ (°C) from *E*′
N_3.0	−14 ± 1	−13 ± 1	50 ± 1
N_2.5	−16 ± 1	−15 ± 1	n.d.
N_2.0	−11 ± 1	−12 ± 1	n.d.
N_1.5	−14 ± 1	−14 ± 1	n.d.

a n.d. – not detected. *T*_m_ could not be detected as the sample broke during heating.

### 3.5. Mechanical properties of PCUU nonwovens

Mechanical analysis of electrospun PCUU nonwovens was conducted to assess their physical properties, such as strength, flexibility, and temperature-dependent behavior, providing critical insights into their suitability for specific applications. Performed analysis helps in understanding how HS content impacts the mechanical performance of the PCUUs.

**Fig. 5:**
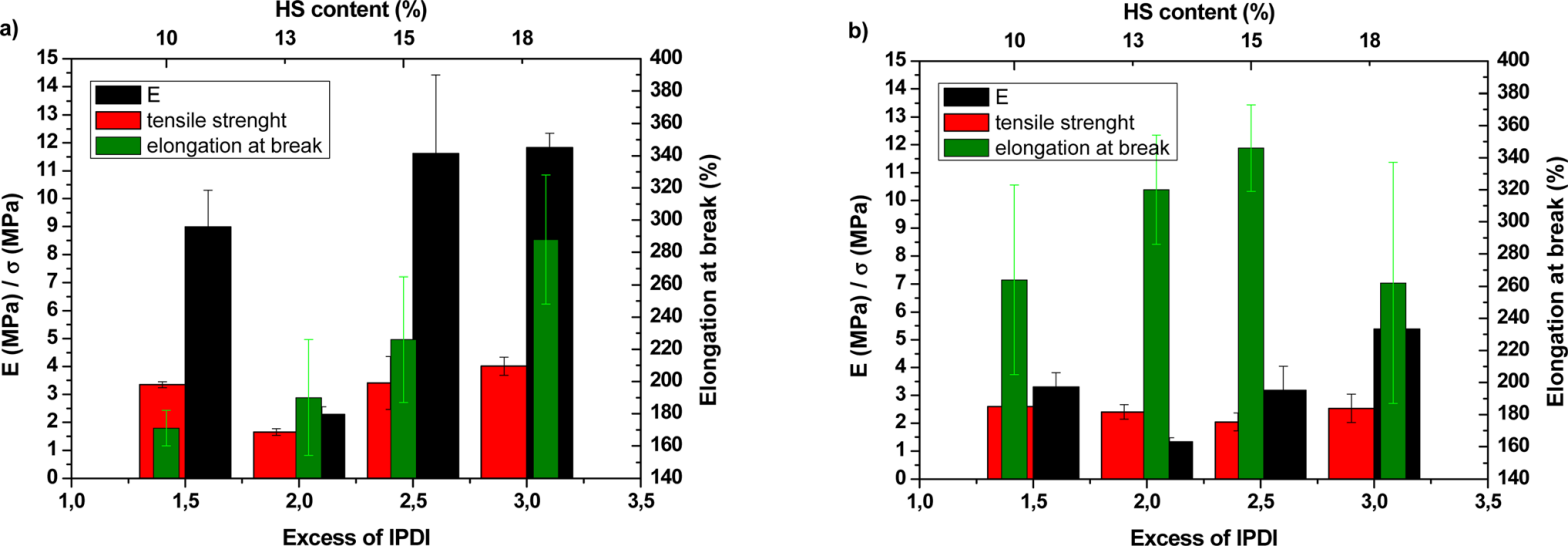
Mechanical properties of PCCU nonwovens at (a) RT and (b) 37 °C.

The analysis of mechanical properties (Fig. 5 and Table S4 in ESI†) conducted at room temperature (RT) revealed that the elongation at break increased with the higher IPDI content. Furthermore, an increase in tensile strength and Young's modulus (*E*) is a result of the higher content of rigid cycloaliphatic groups of hard segments and physical crosslinking among them.

At 37 °C, values of tensile strength and Young's modulus are lower than at RT, which is related most probably to partial melting of the soft segments (OCD), as well as lower or lack of inter-hydrogen bonding between polymer chains. Furthermore, significantly lower mechanical properties of N_2.0 were observed and most probably related to the inhomogeneous structure (morphology) of nonwoven, which contained very big pours between fibers (visible by SEM, Fig. 2). Surprisingly, N_1.5 at both temperatures has shown relatively high tensile strength. This could be assigned to the high amount of crystalline phase, which is a result of the high content of easily crystallizable soft segments and higher regularity in the polymer structure resulted from the lower content of asymmetric IPDI.

## 4. Conclusions

In the presented study we have investigated the influence of HS content on the morphology, thermal, and mechanical properties of electrospun PCUUs. Obtained nonwovens were characterized by a surface porosity in the range of 50 to 57% and a fiber diameter in the range of 0.59 to 0.71 μm. The sample with the highest content of HS (N_3.0) was characterized by higher mechanical properties in comparison to other samples. Furthermore, the highest analyzed content of HS (urea and urethane bonds) caused physical crosslinking, confirmed by the DMTA measurement. We have shown that depending on the HS content, various properties can be achieved, which can be adjusted to the specific and application-related needs.

## Conflicts of interest

There are no conflicts to declare.

## Supplementary Material

RA-014-D4RA01726A-s001
