# Supplementary material for: Influence of the hard segments content on the properties of electrospun aliphatic poly(carbonate-urethane-urea)s
Source: RSC Adv. 2024 May 15;14(22):15766–75. doi: 10.1039/d4ra01726a (PMC11094699; doi:10.1039/d4ra01726a)
Supplement: RA-014-D4RA01726A-s001 [file RA-014-D4RA01726A-s001.pdf]

Electronic Supplementary Material (ESI) for RSC Advances.
This journal is © The Royal Society of Chemistry 2024

Supporting Information

for

Influence of the hard segments content on the properties of electrospun aliphatic poly(carbonate-urethane-urea)s

Karolina Rolińska[1,2,3], Hadi Bakhshi[4], Maria Balk[5], Paweł Parzuchowski[1], Magdalena Mazurek-Budzyńska[1]*

[1] Faculty of Chemistry, Warsaw University of Technology, Noakowskiego 3, 00-664 Warsaw, Poland;

[2] Faculty of Chemistry, University of Warsaw, Pasteura 1, 02-093 Warsaw, Poland;

[3] Łukasiewicz Research Network – Industrial Chemistry Institute, Rydygiera 8, 01-793 Warsaw, Poland

[4] Department of Life Science and Bioprocesses, Fraunhofer Institute for Applied Polymer Research IAP, Geiselbergstraße 69, 14476 Potsdam, Germany;

[5] Institute of Active Polymers, Helmholtz-Zentrum Hereon, Kantstraße 55, 14513 Teltow, Germany;

* magdalena.budzynska@pw.edu.pl – corresponding author

Procedure of the synthesis of oligo(decamethylene carbonate) diol (OCD)

The 56.5 g of 1,10-decanediol (0.32 mol), 150.0 g of E_BMC_8 (0.52 mol) and K2CO3 (0.005 mol.%) were placed in a 500 cm3 three-necked flask equipped with a magnetic stirrer, thermometer, and distillation condenser. Then, approximately 60 cm3 of 1,4-dioxane was added. The temperature of the mixture was gradually increased from 100 °C to 160 °C. The progress of the reaction was monitored by measuring the refractive index of the distillate and carried out until no methanol was observed in the distillate (around 14 h). Afterwards, the reaction was continued under reduced pressure at 160 °C (for an additional 4 h), while the 1,10-decanediol and residual 1,4-dioxane were removed. The obtained product was dissolved in dichloromethane and washed six times with demineralized water to remove the catalyst. Afterwards, the solvent and residuals of water were removed under reduced pressure. The synthesized product was characterized by ^{1}H NMR and FTIR spectroscopy. Based on ^{1}H NMR, the number average molar mass was calculated to be equal 3000 g·mol^{-1}.

OCD:

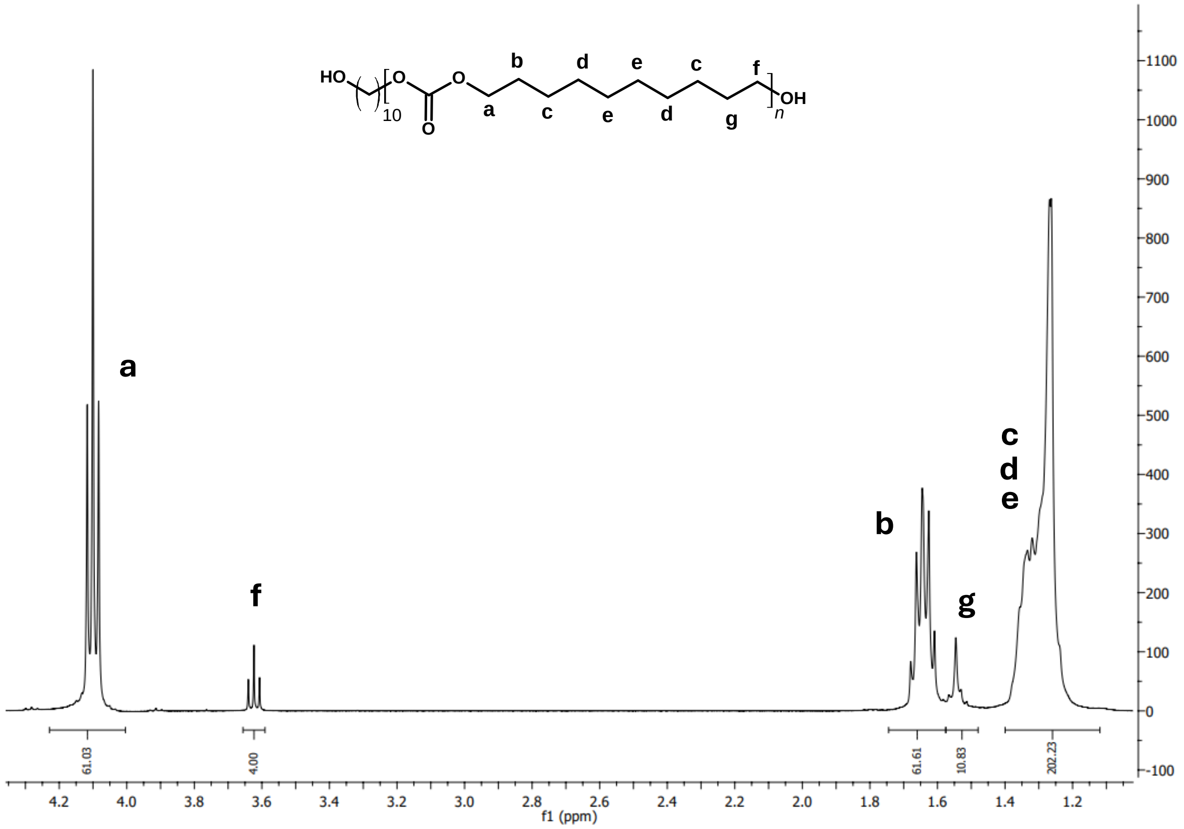

Figure S1. ^{1}H NMR spectrum of OCD.

^{1}H NMR: ($CDCl_3$, 400MHz): δ (ppm)= 4.10 (t, 4H, C(O)OC<u>H₂</u>), 3.62 (t, 4H, C<u>H₂</u>OH), 1.65

(m, 4H, $HOC\underline{H_2}CH_2CH_2CH_2CH_2$), 1.55 (m, 4H, $HOCH_2C\underline{H_2}CH_2CH_2CH_2$), 1.53 (m, 4H, $OCH_2CH_2C\underline{H_2}CH_2CH_2$), 1.32 (m, 4H, $OCH_2CH_2CH_2C\underline{H_2}CH_2$), 1.27 (m, 4H, $OCH_2CH_2CH_2CH_2C\underline{H_2}$).

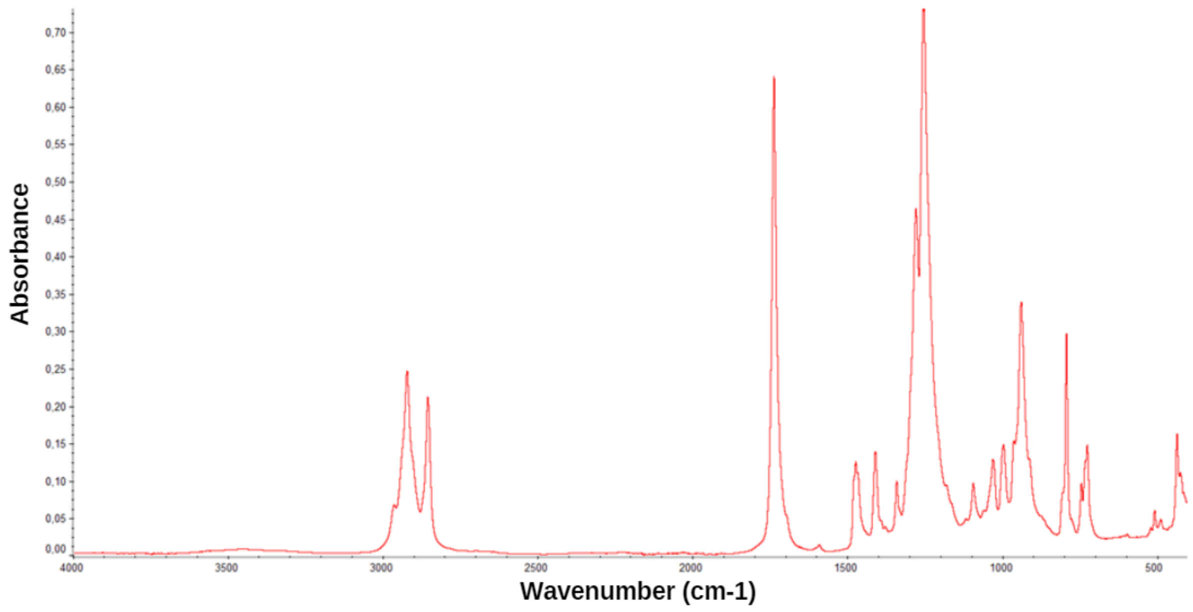

Figure S2. FTIR (ATR) spectrum of OCD.

FTIR (ATR): 3450, 2920, 2850, 1740, 1470, 1400, 1340, 1280, 1250, 1030, 940, 790 cm^{-1}.

Specimens were named accordingly: **PCUU_X** where **X** means the molar excess of the IPDI used for the synthesis. For example, **PCUU_1.5** means that the film was obtained from OCD and IPDI in the molar ratio of 1:1.5

Table S1. The mount of reagents used in the syntheses of the PCUUs.

PCUU	N_3	N_2.5	N_2	N_1.5
**OCD**	20.00 g	20.00 g	20.02 g	20.00 g
**IPDI**	4.40 g	3.70 g	2.95 g	2.24 g

FTIR analysis of prepolymers:

The region 3200-3800 cm^{-1} is assigned to N-H stretching bands. The broad absorption bands 2800–3000 cm^{-1} correspond to the stretching vibration of CH_2 groups. At 2260 cm^{-1} is assigned to NCO stretching bands. In the range of 1620–1780 cm^{-1} the bands of carbonyl groups are

present representing the following groups: amide I (non-hydrogen bonded urethane around 1700 cm^{-1} and hydrogen-bonded urethane around 1690 cm^{-1}), carbonate (hydrogen-bonded around 1720 cm^{-1} and non-hydrogen bonded around 1740 cm^{-1}). Amide II bands from urethane could also be identified in the spectra and located around 1520-1565 cm^{-1}.

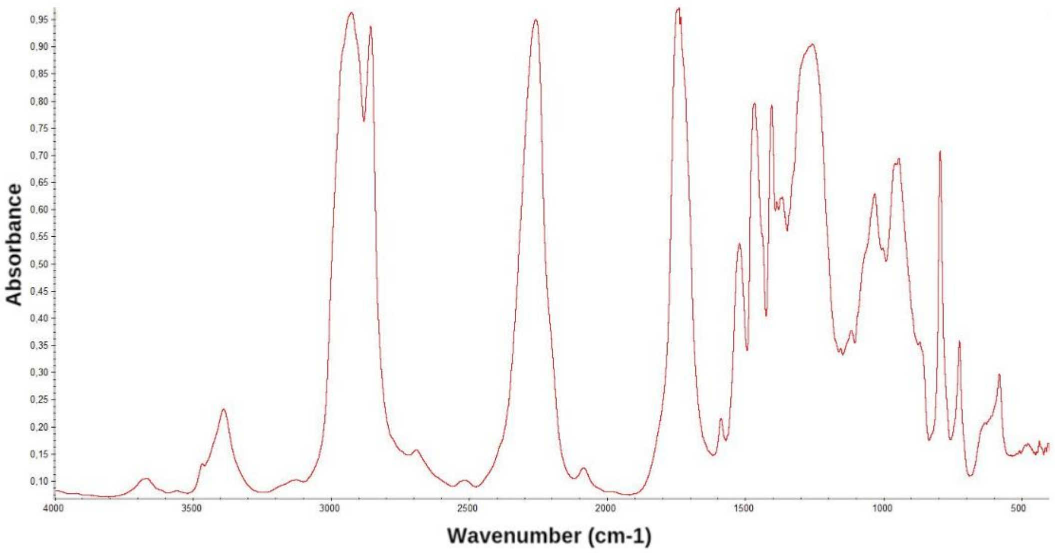

Figure S3. FTIR spectra of PCUU_3 carbonate-urethane diisocyanate prepolymer.

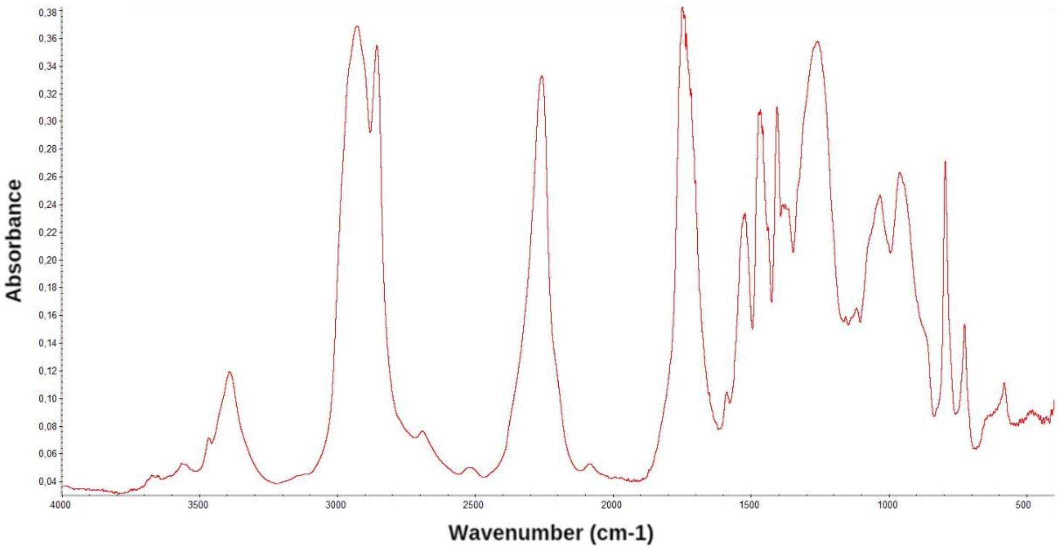

Figure S4. FTIR spectra of PCUU_2.5 carbonate-urethane diisocyanate prepolymer.

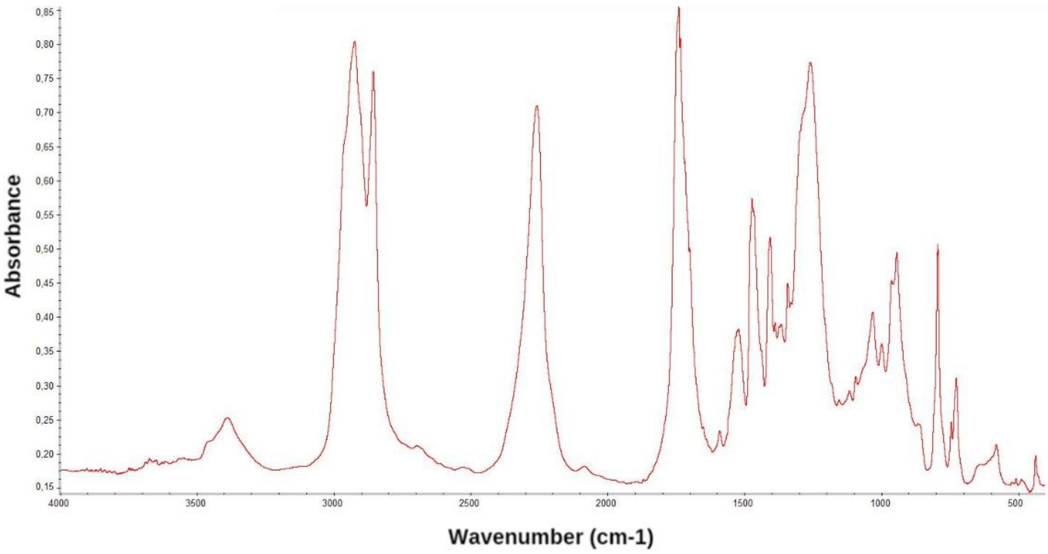

Figure S5. FTIR spectra of PCUU_2 carbonate-urethane diisocyanate prepolymer.

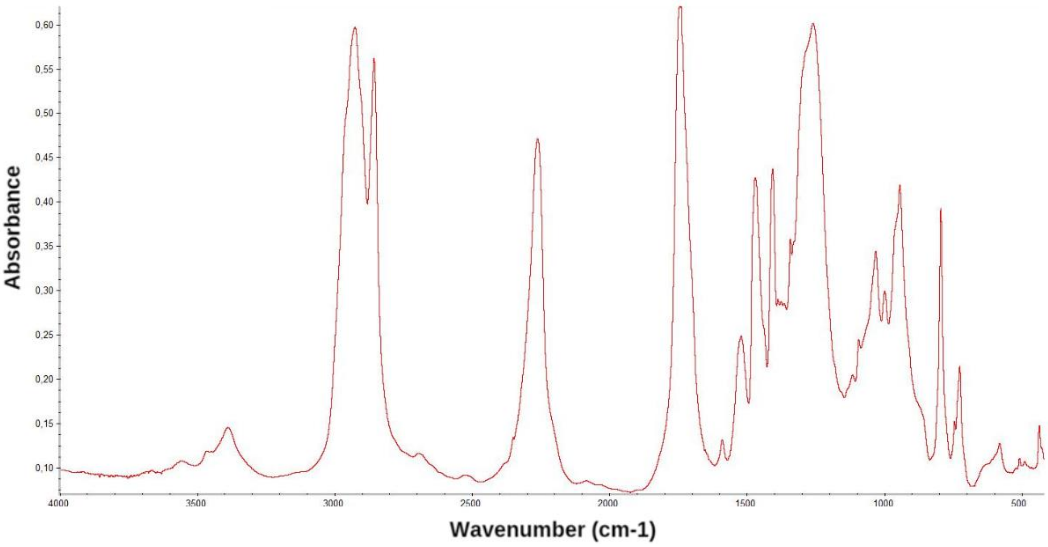

Figure S6. FTIR spectra of PCUU_1.5 carbonate-urethane diisocyanate prepolymer.

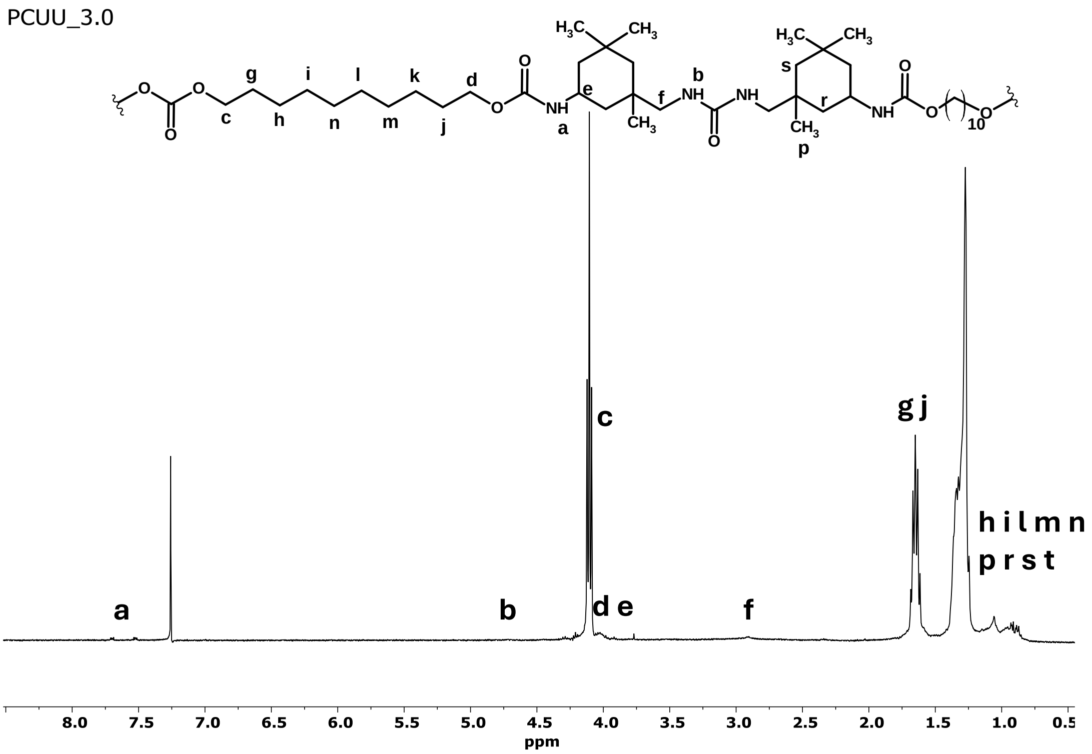

Figure S7. ^{1}H-NMR ($CDCl_3$, 400MHz) spectrum of PCUU_3.

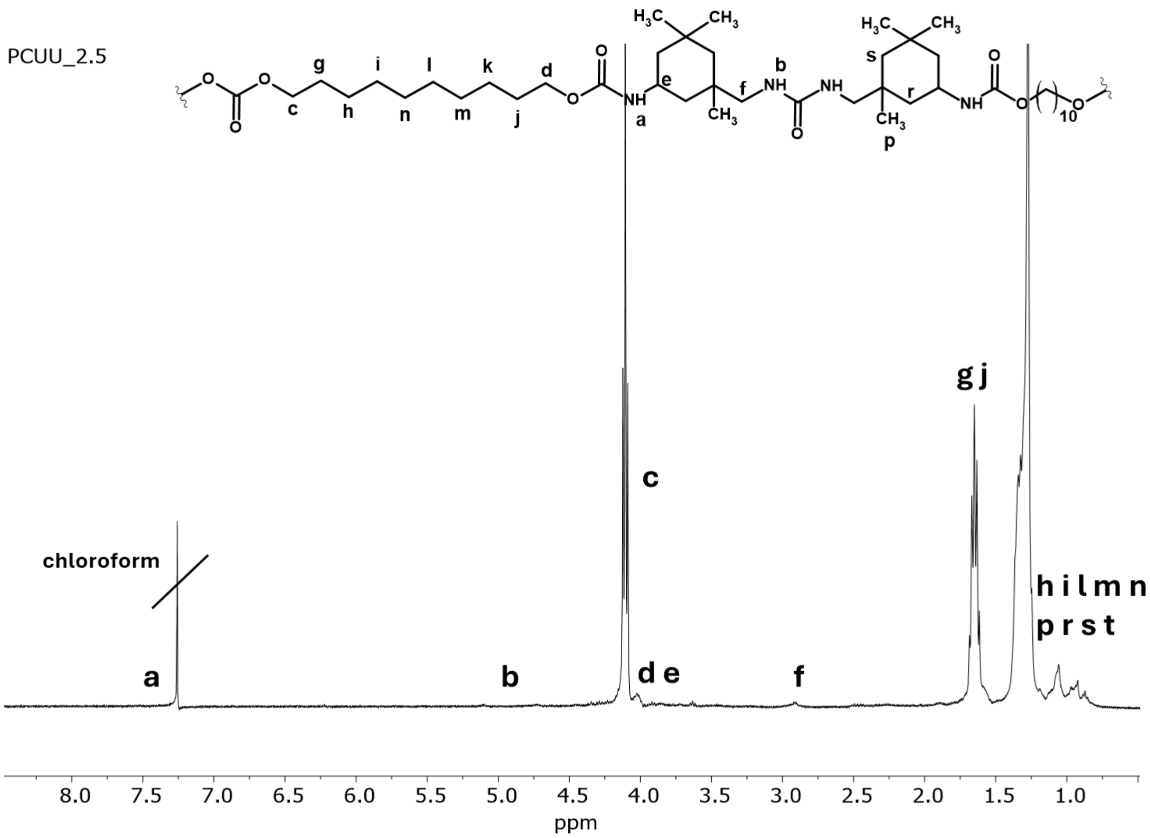

Figure S8. ^{1}H-NMR ($CDCl_3$, 400MHz) spectrum of PCUU_2.5.

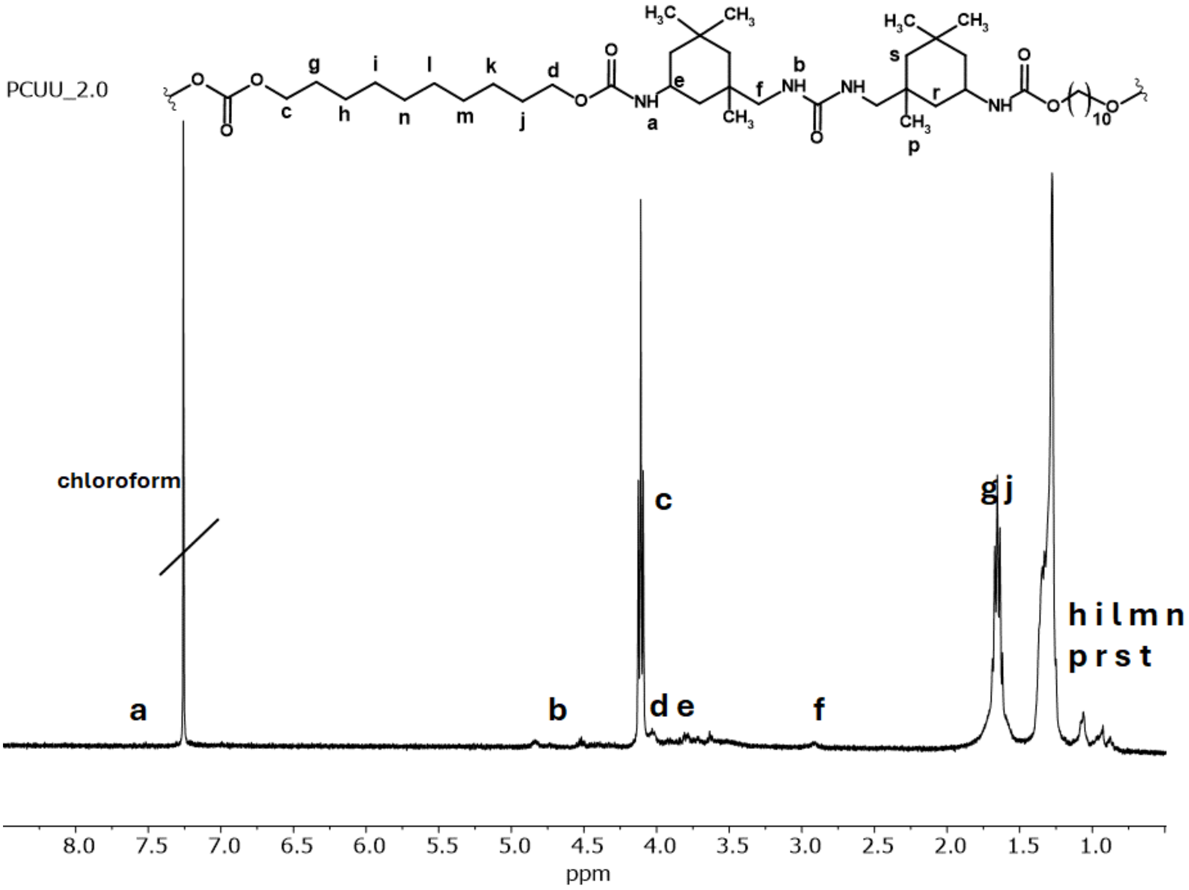

Figure S9. ^{1}H-NMR ($CDCl_3$, 400MHz) spectrum of PCUU_2.

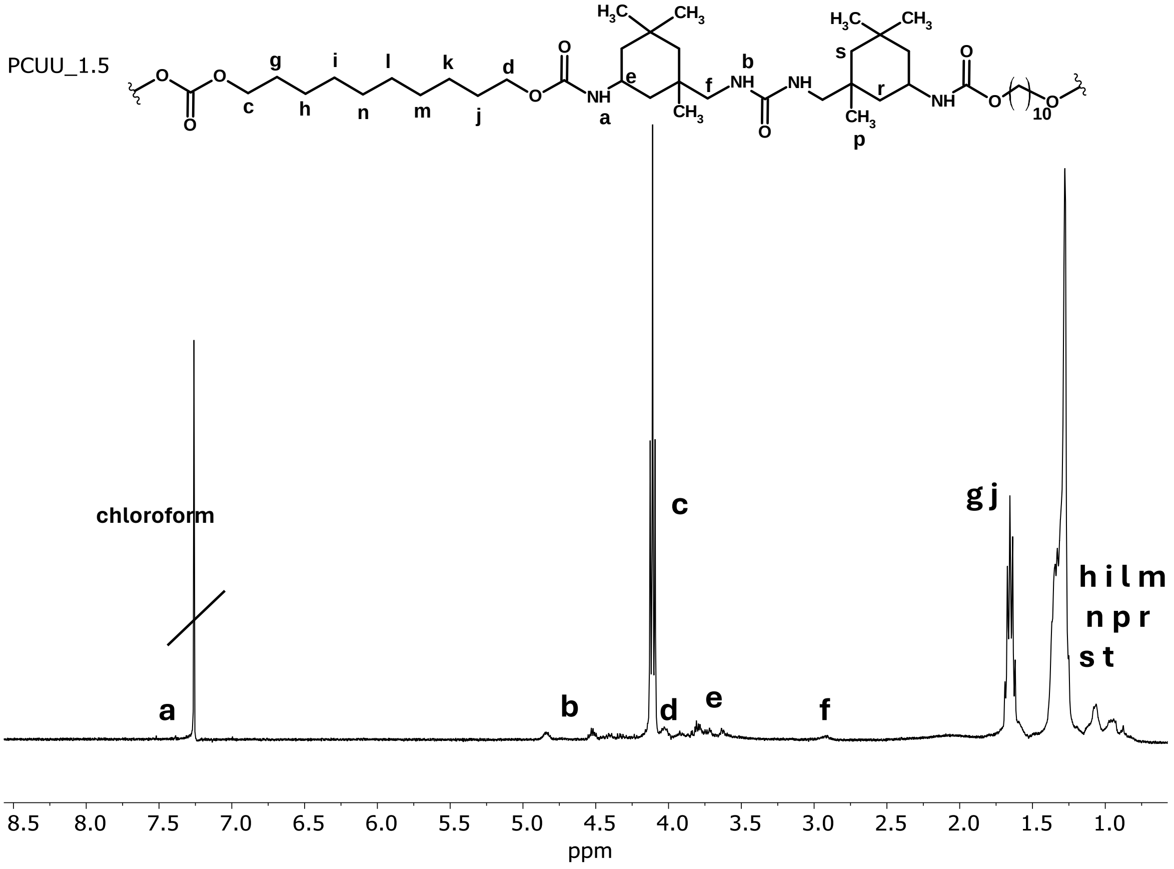

Figure S10. ^{1}H-NMR ($CDCl_3$, 400MHz) spectrum of PCUU_1.5.

FTIR analysis:

The region 3200-3500 cm^{-1} is assigned to N-H stretching bands. The broad absorption bands 2800–3000 cm^{-1} correspond to the stretching vibration of CH_2 groups. In the range of 1620–1780 cm^{-1} the bands of carbonyl groups are present representing the following groups: amide I (non-hydrogen bonded urethane around 1700 cm^{-1} and hydrogen-bonded urethane around 1690 cm^{-1}), carbonate (hydrogen-bonded around 1720 cm^{-1} and non-hydrogen bonded around 1740 cm^{-1}), and hydrogen-bonded urea carbonyl band at nearly 1630 cm^{-1}. Amide II bands from urethane and urea could also be identified in the spectra and located around 1520-1565 cm^{-1}.

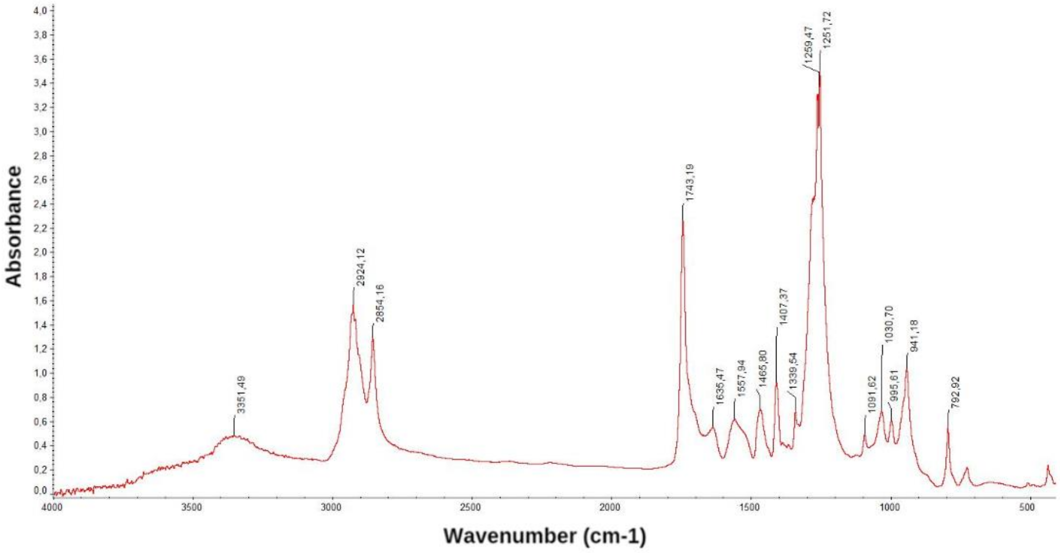

Figure S11. FTIR spectra of PCUU_3.

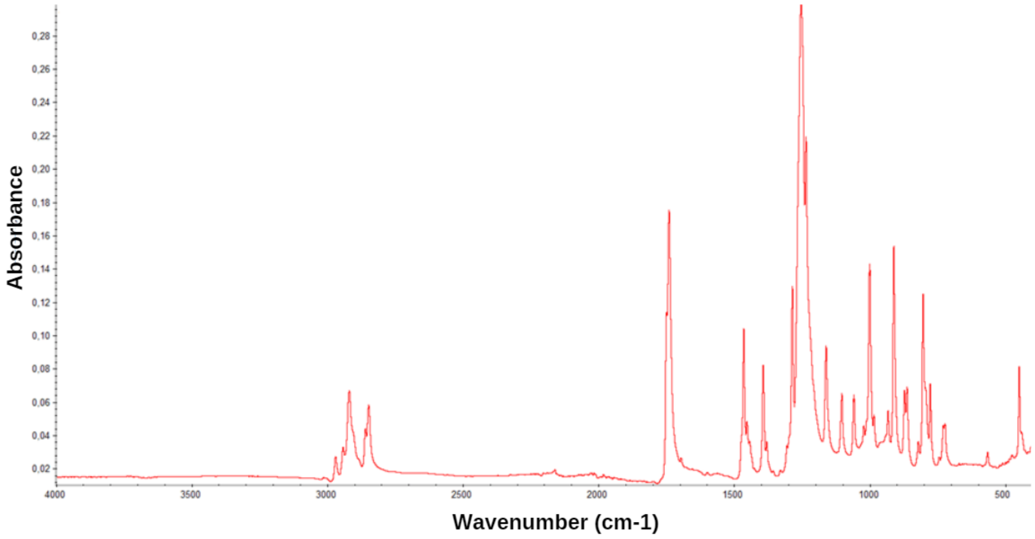

Figure S12. ATR-FTIR spectrum of PCUU_3.

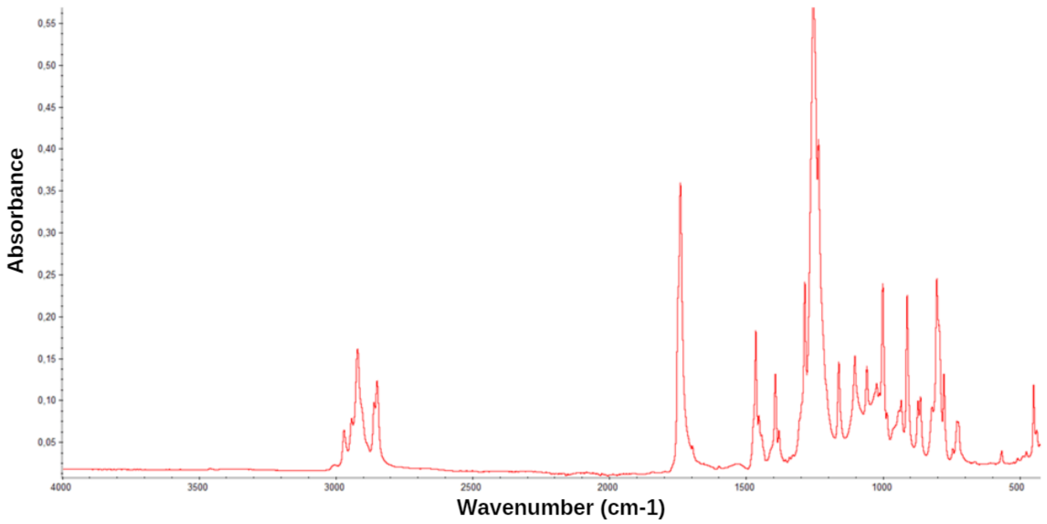

Figure S13 ATR-**.** FTIR spectrum of PCUU_2.5.

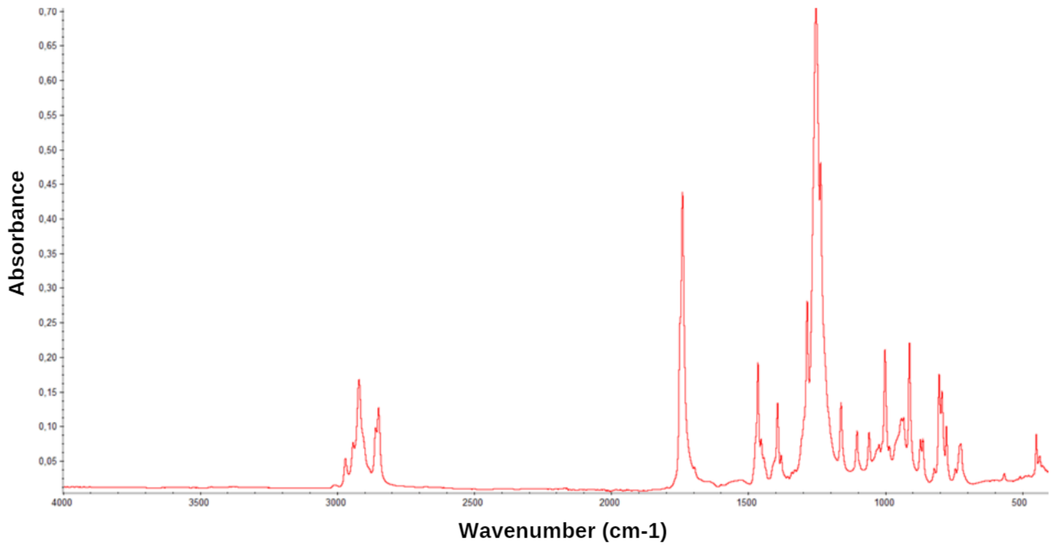

Figure S14. ATR-FTIR spectrum of PCUU_2.

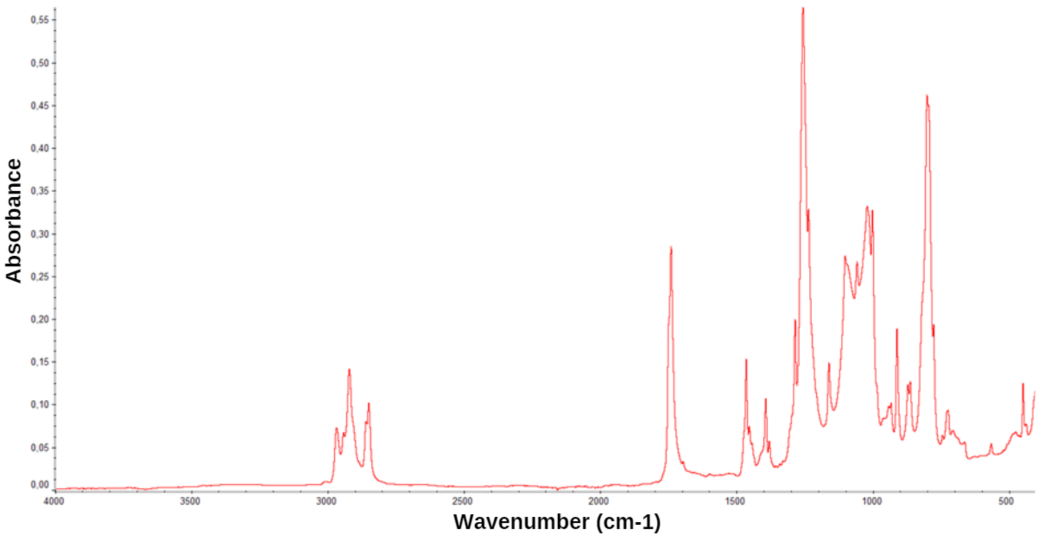

Figure S15 ATR-FTIR spectrum of PCUU_1.5.

KR-20 Printed: Tuesday, February 01, 2022 12:18:24
C:\Users\Viscotek\Documents\OmniSEC Data\Viscotek\2021-12\2022-01-28_12;16;42_KR-20_01.vdt

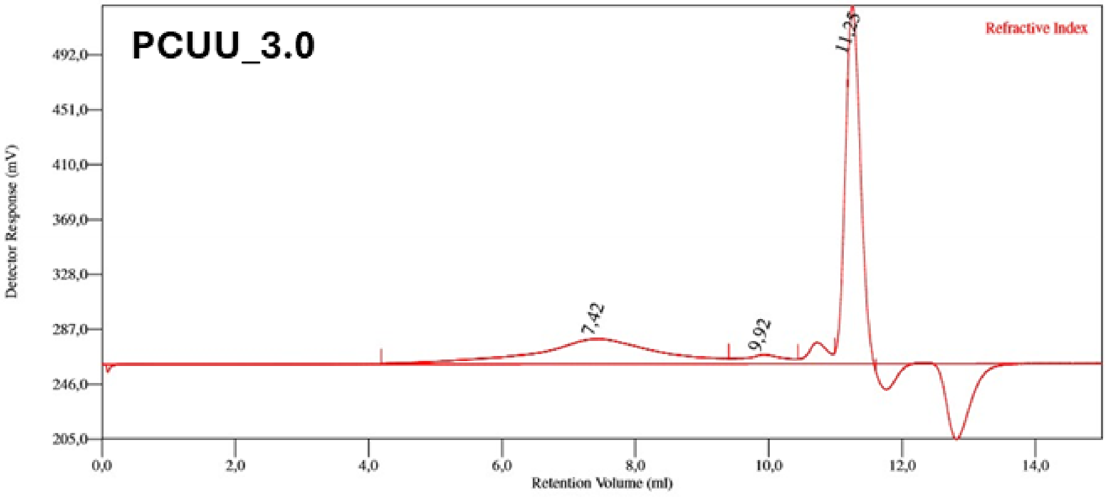

Conventional Calibration - Homopolymers : Results

Peak RV - (ml)	7,423	9,923	11,247
Mn - (Daltons)	23 546	412	0
Mw - (Daltons)	2,008 e 6	685	0
Mz - (Daltons)	1,046 e 8	1 005	0
Mp - (Daltons)	62 675	537	0
Mw / Mn	85,298	1,663	0,000
Percent Above Mw: 0	100,000	100,000	0,000
Percent Below Mw: 0	0,000	0,000	0,000
Mw 10.0% Low	5 064	151	0
Mw 10.0% High	1,889 e 7	1 684	0
Wt Fr (Peak)	0,888	0,112	0,000
RI Area - (mvml)	39,94	5,04	69,82
UV@240nm Area - (mvml)	0,00	0,00	0,00

Annotation	
Method File	CC_RI_PS_2021-09_z_dnia_2022-01-05-0003.vcm
Limits File	
Date Acquired	Jan 28, 2022 - 12:16:42
Solvent	Chlorek metylenu
Acquisition Operator	admin : Administrator
Calculation Operator	admin : Administrator
Column Set	Jordi DVB Mixed Bed
System	QDA MB
Flow Rate - (ml/min)	1,000
Inj Volume - (ul)	120,0
Volume Increment - (ml)	0,00333
Detector Temp. - (deg C)	30,0
Column Temp. - (deg C)	30,0
OmniSEC Build Number	467

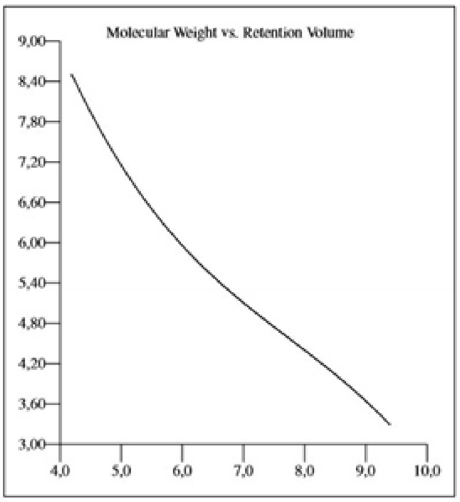

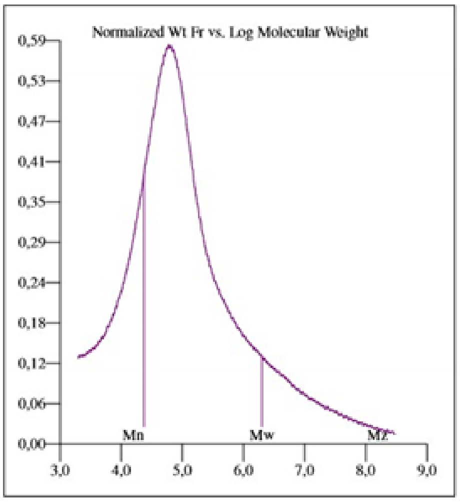

Figure S16. GPC curves of PCUU_3.0.

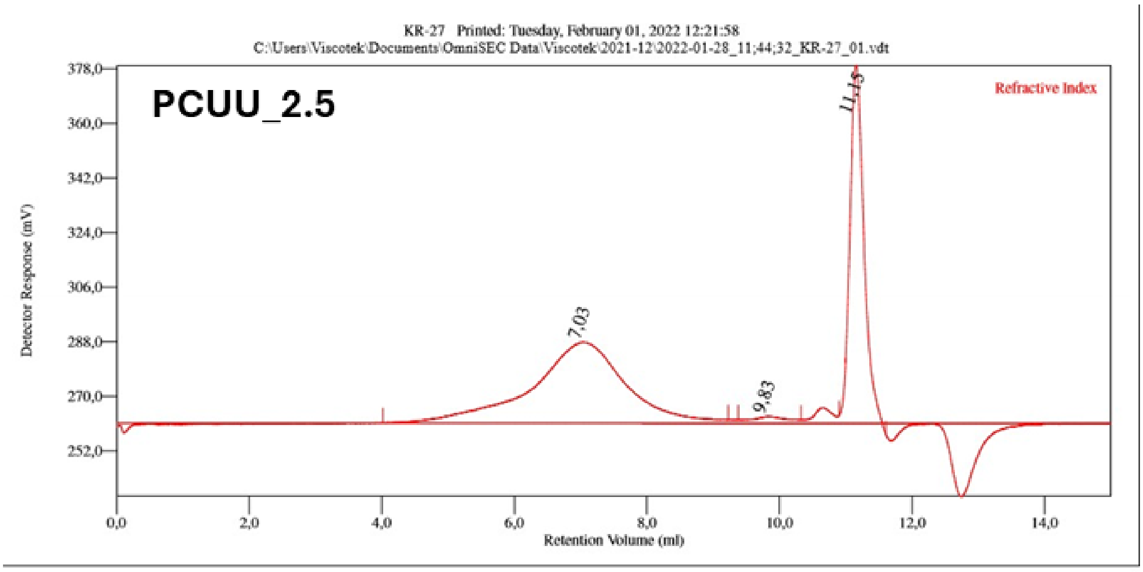

Conventional Calibration - Homopolymers : Results

Peak RV - (ml)	7,033	9,827	11,150
Mn - (Daltons)	56 286	406	0
Mw - (Daltons)	1,674 e 6	627	0
Mz - (Daltons)	1,338 e 8	881	0
Mp - (Daltons)	107 886	553	0
Mw / Mn	29,737	1,546	0,000
Percent Above Mw: 0	100,000	100,000	0,000
Percent Below Mw: 0	0,000	0,000	0,000
Mw 10.0% Low	15 608	157	0
Mw 10.0% High	1,415 e 7	1 468	0
Wt Fr (Peak)	0,968	0,032	0,000
RI Area - (mvml)	43,51	1,43	28,77
UV@240nm Area - (mvml)	0,00	0,00	0,00

Annotation	
Method File	CC_RI_PS_2021-09_z_dnia_2022-01-05-0003.vcm
Limits File	
Date Acquired	Jan 28, 2022 - 11:44:32
Solvent	Chlorek metylenu
Acquisition Operator	admin : Administrator
Calculation Operator	admin : Administrator
Column Set	Jordi DVB Mixed Bed
System	QDA MB
Flow Rate - (ml/min)	1,000
Inj Volume - (ul)	50,0
Volume Increment - (ml)	0,00333
Detector Temp. - (deg C)	30,0
Column Temp. - (deg C)	30,0
OmniSEC Build Number	467

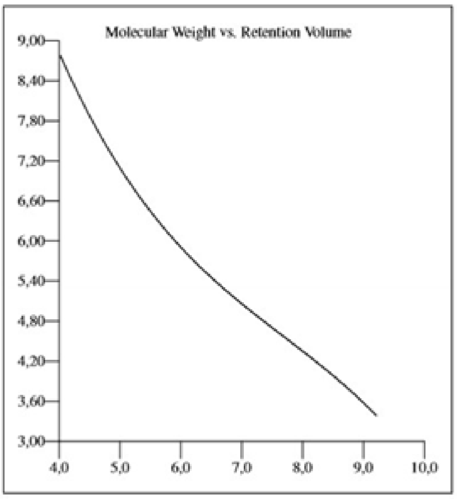

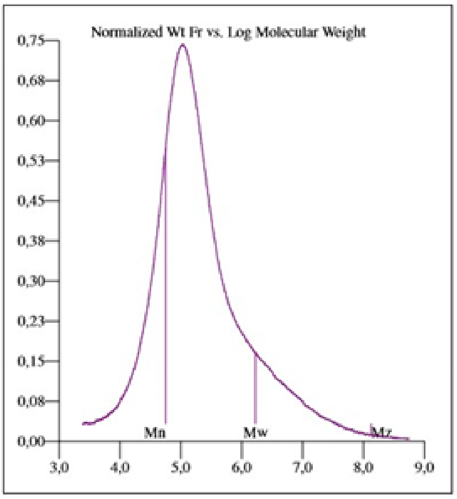

Figure S17. GPC curves of PCUU_2.5.

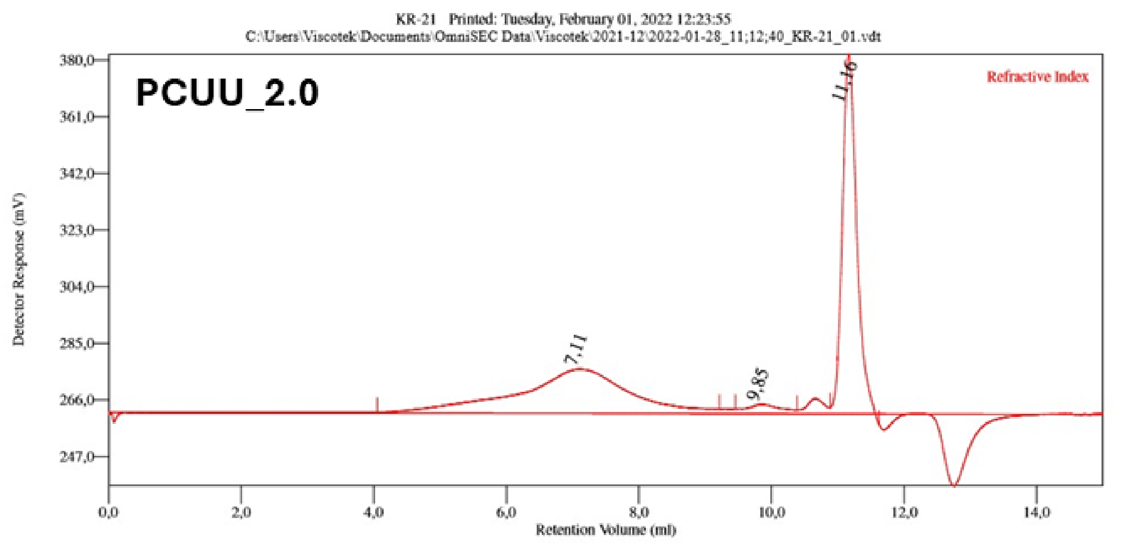

Conventional Calibration - Homopolymers : Results

Peak RV - (ml)	7,107	9,850	11,160
Mn - (Daltons)	43 746	366	0
Mw - (Daltons)	2,665 e 6	556	0
Mz - (Daltons)	1,303 e 8	759	0
Mp - (Daltons)	96 554	535	0
Mw / Mn	60,921	1,520	0,000
Percent Above Mw: 0	100,000	100,000	0,000
Percent Below Mw: 0	0,000	0,000	0,000
Mw 10.0% Low	10 586	138	0
Mw 10.0% High	2,427 e 7	1 250	0
Wt Fr (Peak)	0,937	0,063	0,000
RI Area - (mvml)	28,71	1,95	30,10
UV@240nm Area - (mvml)	0,00	0,00	0,00

Annotation	
Method File	CC_RI_PS_2021-09_z_dnia_2022-01-05-0003.vcm
Limits File	
Date Acquired	Jan 28, 2022 - 11:12:40
Solvent	Chlorek metylenu
Acquisition Operator	admin : Administrator
Calculation Operator	admin : Administrator
Column Set	Jordi DVB Mixed Bed
System	QDA MB
Flow Rate - (ml/min)	1,000
Inj Volume - (ul)	50,0
Volume Increment - (ml)	0,00333
Detector Temp. - (deg C)	30,0
Column Temp. - (deg C)	30,0
OmniSEC Build Number	467

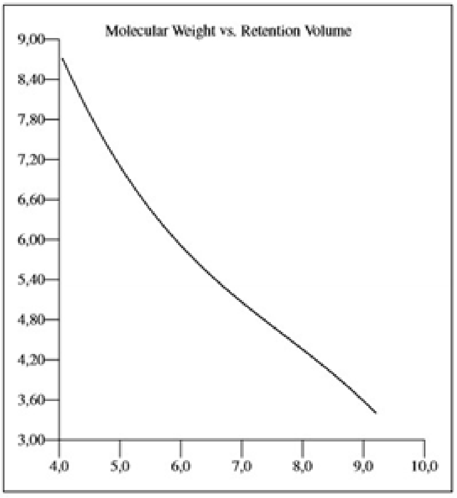

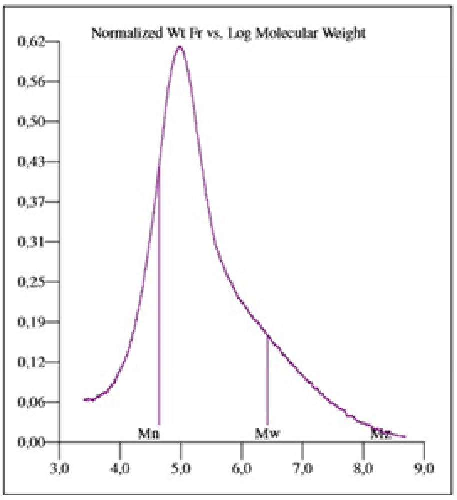

Figure S18. GPC curves of PCUU_2.0.

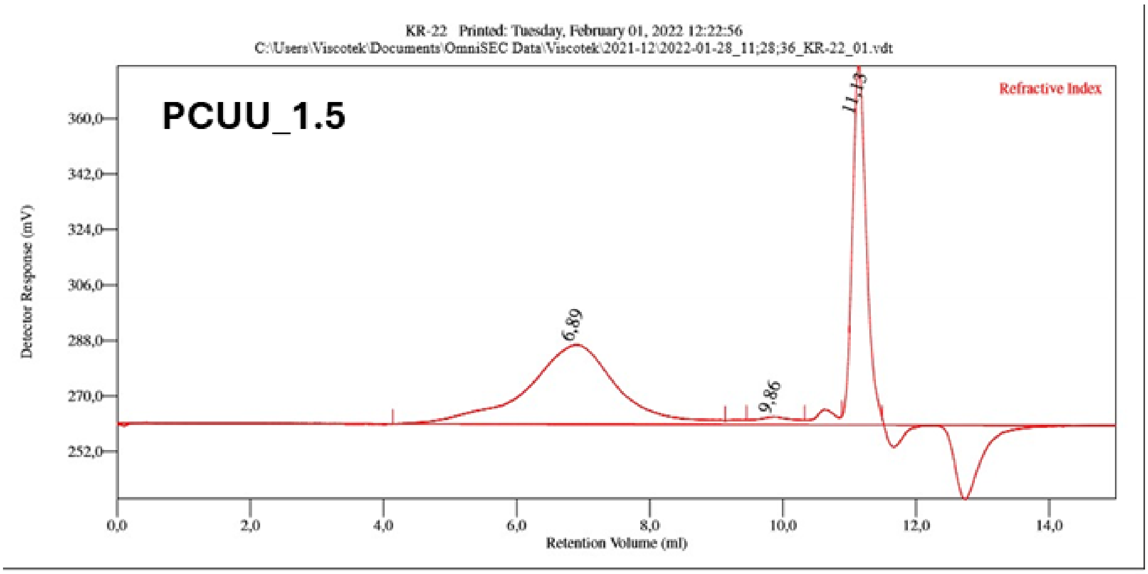

Conventional Calibration - Homopolymers : Results

Peak RV - (ml)	6,893	9,860	11,133
Mn - (Daltons)	64 588	363	0
Mw - (Daltons)	1,169 e 6	539	0
Mz - (Daltons)	5,951 e 7	733	0
Mp - (Daltons)	135 223	488	0
Mw / Mn	18,106	1,483	0,000
Percent Above Mw: 0	100,000	100,000	0,000
Percent Below Mw: 0	0,000	0,000	0,000
Mw 10,0% Low	18 087	147	0
Mw 10,0% High	8,029 e 6	1 206	0
Wt Fr (Peak)	0,959	0,041	0,000
RI Area - (mvml)	40,22	1,71	28,45
UV@240nm Area - (mvml)	0,00	0,00	0,00

Annotation	
Method File	CC_RI_PS_2021-09_z_dnia_2022-01-05-0003.vcm
Limits File	
Date Acquired	Jan 28, 2022 - 11:28:36
Solvent	Chlorek metylenu
Acquisition Operator	admin : Administrator
Calculation Operator	admin : Administrator
Column Set	Jordi DVB Mixed Bed
System	QDA MB
Flow Rate - (ml/min)	1,000
Inj Volume - (ul)	50,0
Volume Increment - (ml)	0,00333
Detector Temp. - (deg C)	30,0
Column Temp. - (deg C)	30,0
OmniSEC Build Number	467

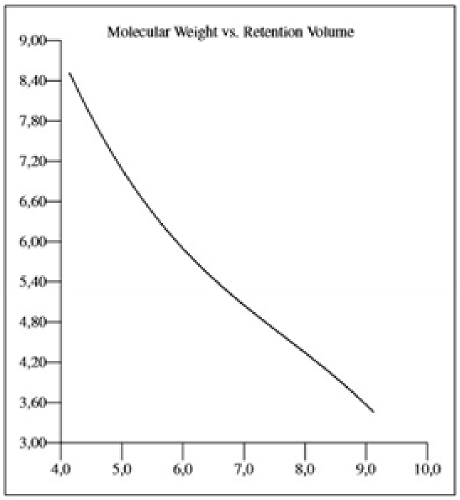

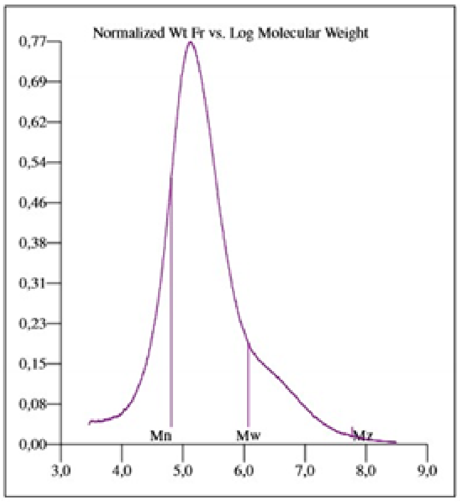

Figure S19. GPC curves of PCUU_1.5.

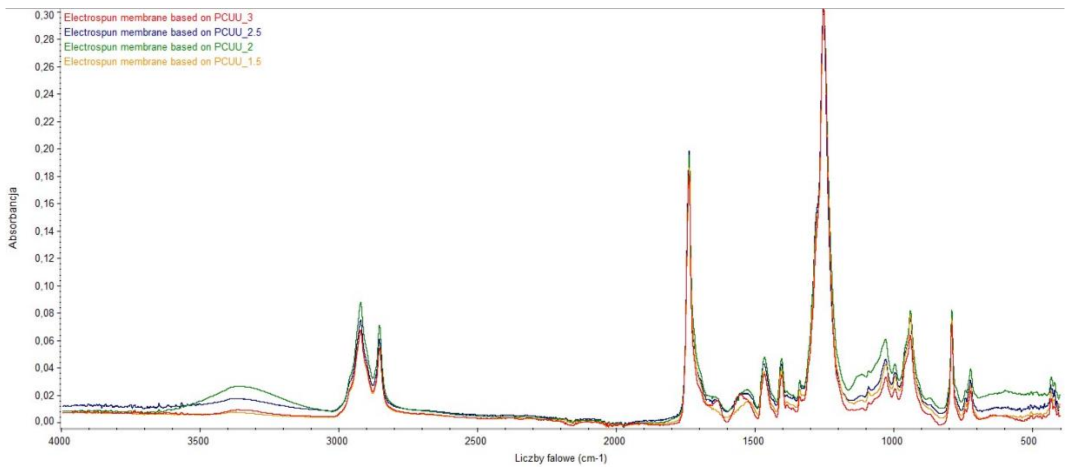

Figure S20. ATR-FTIR spectrum of spectra of N_3 (red line), N_2.5 (purple line), N_2 (green line), and N_1.5 (yellow line).

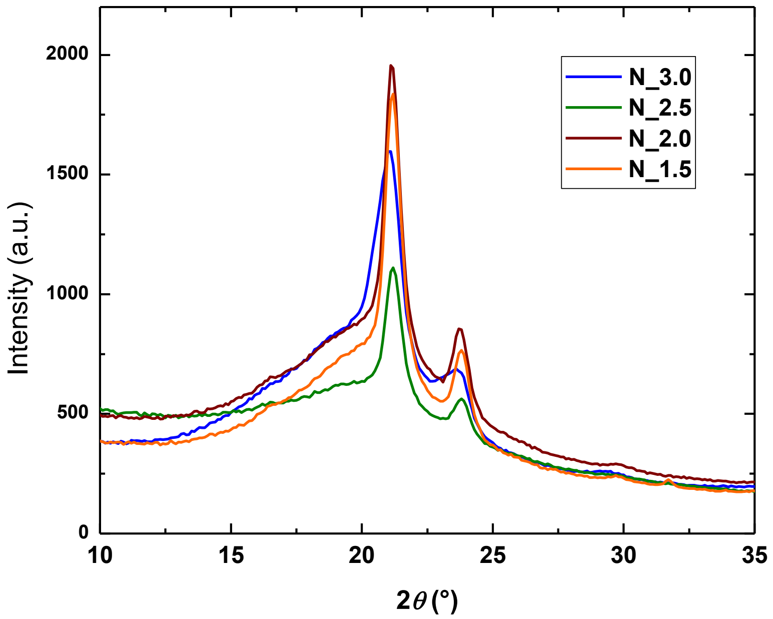

Figure S21. WAXS curves of scattering angle 2θ (°) for PCUU nonwovens.

Table S3. Average degree of crystallinity (DOC) and average crystal size (l_c) of PCUU nonwovens determined by WAXS.

Sample	HS (wt.%)	Average DOC (%)	Average l_c (nm)
**N_3.0**	18 ± 1	28.4 ± 0.8	9.1 ± 0.2
**N_2.5**	15 ± 1	29.1 ± 0.3	13.8 ± 0.1
**N_2.0**	13 ± 1	30.6 ± 4.9	15.1 ±0 .3
**N_1.5**	10 ± 1	29.0 ± 0.2	14.4 ± 0.0

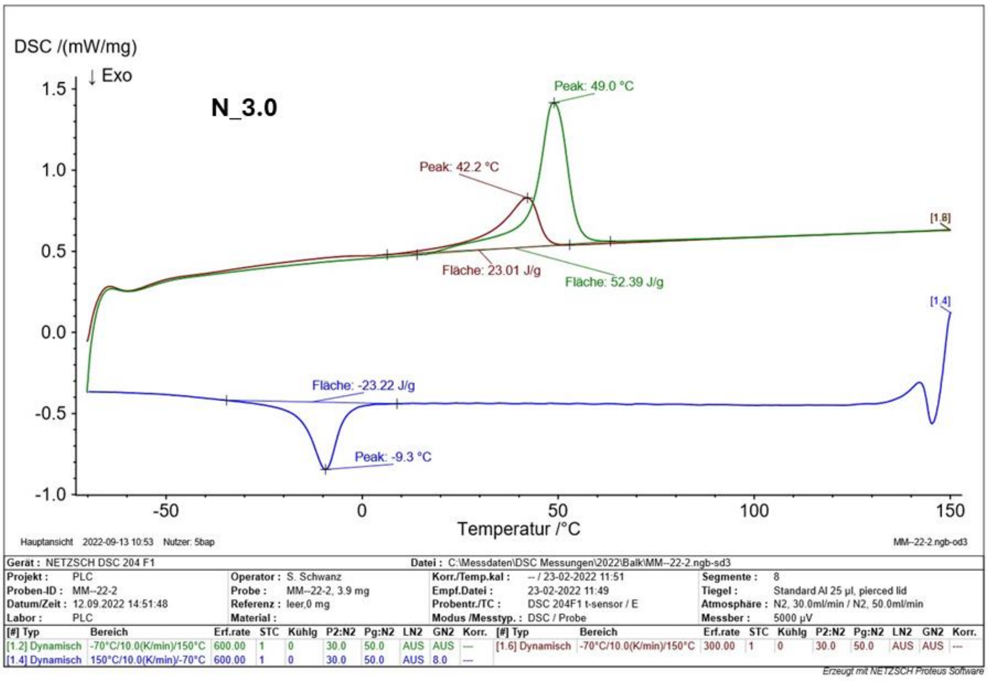

Figure S22. DSC curves of N_3.0.

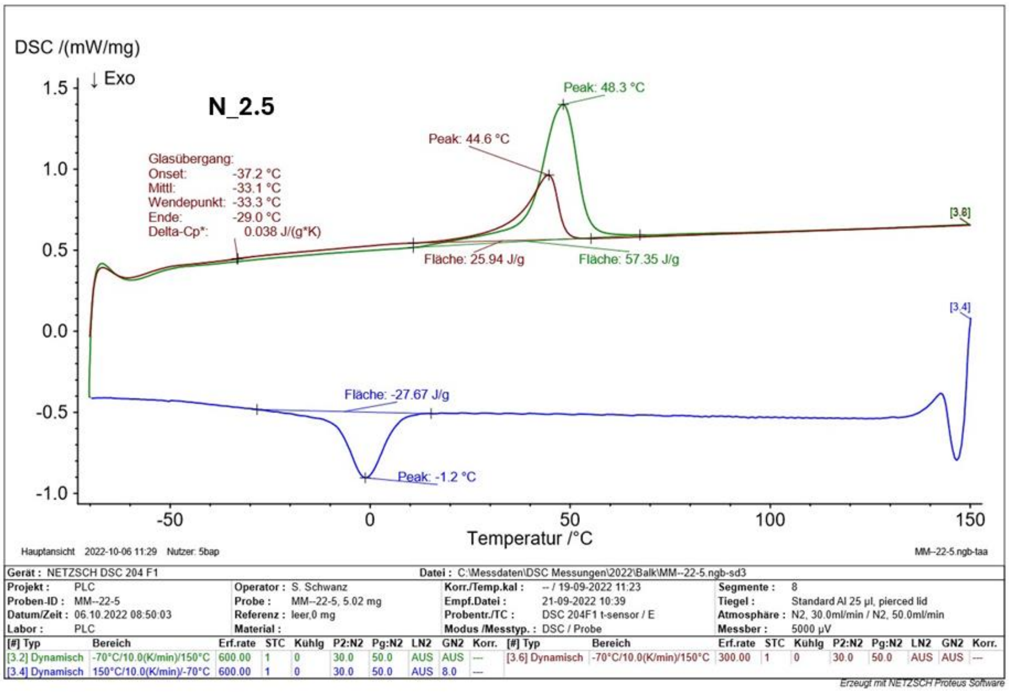

Figure S23. DSC curves of N_2.5.

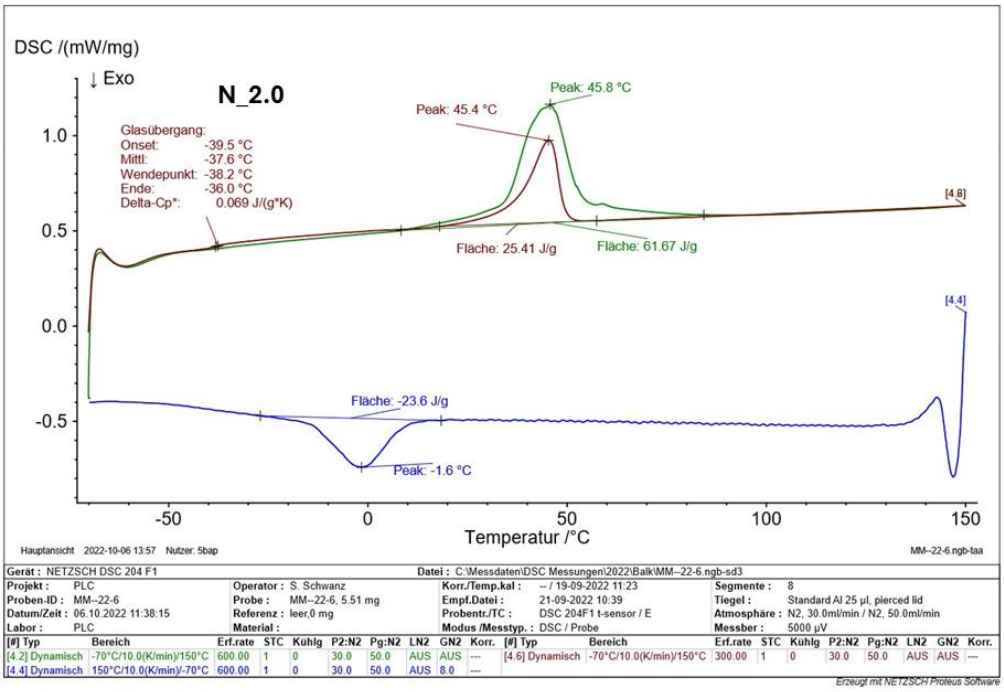

Figure S24. DSC curves of N_2.0.

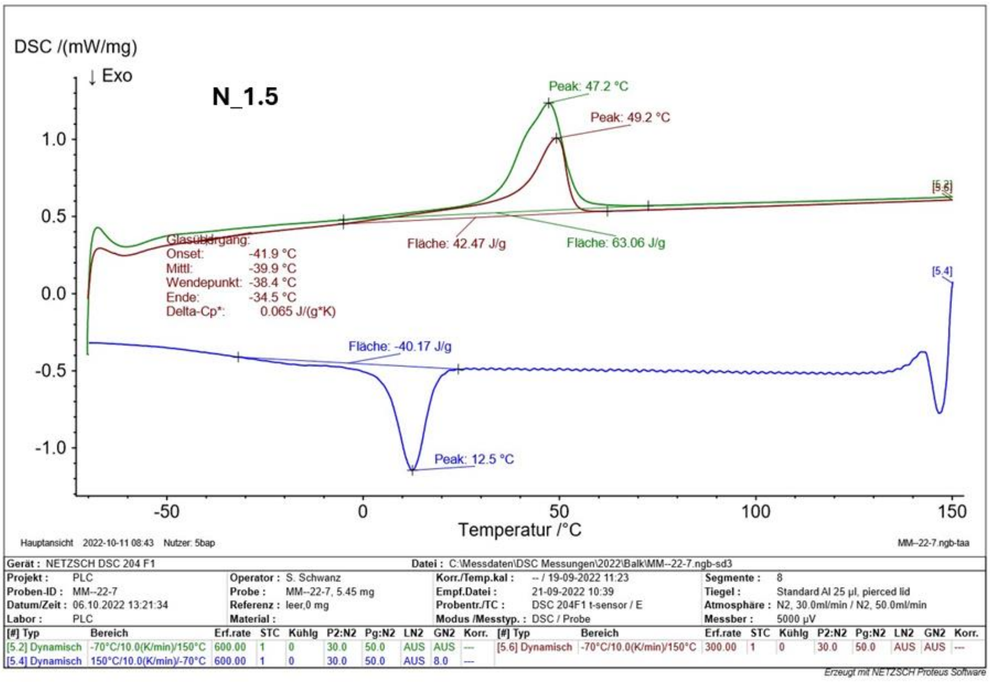

Figure S25. DSC curves of N_1.5.

Table S4. Mechanical properties of PCUU nonwovens at RT and 37 °C.

Temp.	Sample	HS (wt.%)	E (MPa)	σ (MPa)	ε (%)
RT	N_3.0	18 ± 1	11.83 ± 0.52	4.01 ± 0.32	288 ± 40
	N_2.5	15 ± 1	11.61 ± 2.81	3.41 ± 0.95	226 ± 39
	N_2.0	13 ± 1	2.28 ± 0.28	1.65 ± 0.12	190 ± 36
	N_1.5	10 ± 1	8.99 ± 1.31	3.35 ± 0.11	171 ± 11
37°C	N_3.0	18 ± 1	5.39 ± 1.06	2.53 ± 0.51	262 ± 75
	N_2.5	15 ± 1	3.18 ± 0.87	2.04 ± 0.32	346 ± 27
	N_2.0	13 ± 1	1.33 ± 0.14	2.40 ± 0.26	320 ± 34
	N_1.5	10 ± 1	3.30 ± 0.52	2.60 ± 0.20	264 ± 59
